# Many faces of acute bowel ischemia: overview of radiologic staging

**DOI:** 10.1186/s13244-021-00985-9

**Published:** 2021-04-29

**Authors:** Amir H. Davarpanah, Afshar Ghamari Khameneh, Bardia Khosravi, Ali Mir, Hiva Saffar, Amir Reza Radmard

**Affiliations:** 1 Department of Radiology and Imaging Sciences, Emory University School of Medicine, Atlanta, USA; 2 Department of Radiology, Faculty of Medicine, Mashhad University of Medical Sciences, Mashhad, Iran; 3 Department of Radiology, Shariati Hospital, Tehran University of Medical Sciences, 14117, North Kargar St., Tehran, Iran; 4 Department of Surgery, Shariati Hospital, Tehran University of Medical Sciences, Tehran, Iran; 5 Department of Pathology, Shariati Hospital, Tehran University of Medical Sciences, Tehran, Iran

**Keywords:** Acute bowel ischemia, Radiologic staging, Pathologic correlation, Management

## Abstract

Acute bowel ischemia (ABI) can be life threatening with high mortality rate. In spite of the advances made in diagnosis and treatment of ABI, no significant change has occurred in the mortality over the past decade. ABI is potentially reversible with prompt diagnosis. The radiologist plays a central role in the initial diagnosis and preventing progression to irreversible intestinal ischemic injury or bowel necrosis. The most single imaging findings described in the literature are either non-specific or only present in the late stages of ABI, urging the use of a constellation of features to reach a more confident diagnosis. While ABI has been traditionally categorized based on the etiology with a wide spectrum of imaging findings overlapped with each other, the final decision for patient’s management is usually made on the stage of the ABI with respect to the underlying pathophysiology. In this review, we first discuss the pathologic stages of ischemia and then summarize the various imaging signs and causes of ABI. We also emphasize on the correlation of imaging findings and pathological staging of the disease. Finally, a management approach is proposed using combined clinical and radiological findings to determine whether the patient may benefit from surgery or not.

## Key Points

- There is a correlation between pathologic staging and radiologic findings of ABI.
- Reversibility–irreversibility of ABI spans a temporal continuum with areas of overlap.
- Understanding the pathophysiology of ABI can help for better management.
- Management should be based on the latest stage identified.

## Background

Acute bowel ischemia (ABI) is defined as bowel injury associated with sudden interruption of blood supply to small or large intestine in an either segmental or diffuse pattern [1]. Despite low incidence (accounting for only 0.09–0.2% of emergency departments admissions [2]), this is a life-threatening condition with mortality rate reaching 60–80%, even with surgical intervention [3]. In spite of advances in detection and therapeutic options, no significant change has occurred in the mortality of ABI over the past decade [4]. Furthermore, with increasing mean life expectancy, bowel ischemia has turned into one of the most ominous disorders of elderly patients in the clinical practice [5].

Clinical presentation, physical examination and laboratory findings are generally non-specific and do not reliably differentiate bowel ischemia from other abdominal emergencies without imaging [6]. Patients with ABI may present with abdominal pain out of proportion to examination, rebound tenderness, epigastric bruit, vomiting and diarrhea, most of which happening in the late stages of the disease [7]. Elevation of serum lactate levels could be also a late finding in the course of disease. This is true for other laboratory findings including neutrophilic leukocytosis, high anion gap metabolic acidosis, lactate dehydrogenase (LDH) and aspartate aminotransferase (AST) [8].

Duration of insufficient blood flow is the most important prognostic factor which makes timely diagnosis and surgical intervention the cornerstones of improved outcome [9]. Although X-ray (abdominal or upright chest) and abdominal ultrasound play a vital role in the first-line diagnostic workup of acute abdomen, they usually yield non-diagnostic results in case of ABI [10]. Abdominal x-ray can show pneumoperitoneum, caused by bowel perforation in advanced stages of ABI, or even in some instances may show pneumatosis intestinalis in late in the disease course. Abdominal X-ray can also depict bowel distention as a non-specific finding caused by paralytic ileus in early stages of ABI. Abdominal ultrasound might be useful to assess intraperitoneal free fluid or portal venous gas in advanced ABI. Doppler study may also infrequently reveal arterial or venous occlusion in larger vessels.

With a 93% sensitivity and 96% specificity, multidetector computed tomography (MDCT) scan is widely utilized as the first-line imaging choice to confirm and localize ABI, determine its severity and exclude mimickers [11]. MRI is not a common choice for diagnosing ABI in acute clinical settings, and due to its better visualization of thrombosis, inflammatory processes and adhesion bands, it is more frequently used in evaluation of chronic bowel ischemia [10].

ABI tends to develop through different stages with distinctive diagnostic features at each stage [12]. CT scan plays an important role in appropriate triage of patients and early diagnosis of disease at the reversible stage. Thus, radiologists play a key role in the initial diagnosis by determining the location, extent of the disease and identifying the underlying cause, potentially preventing ABI progression to irreversible intestinal injury or bowel necrosis [13].

This review will address various causes and imaging signs of bowel ischemia with emphasizes on correlation of imaging findings and pathological staging of the disease. Finally, a management approach is proposed using combined clinical and radiological findings.

## Etiology

**Fig. 1:**
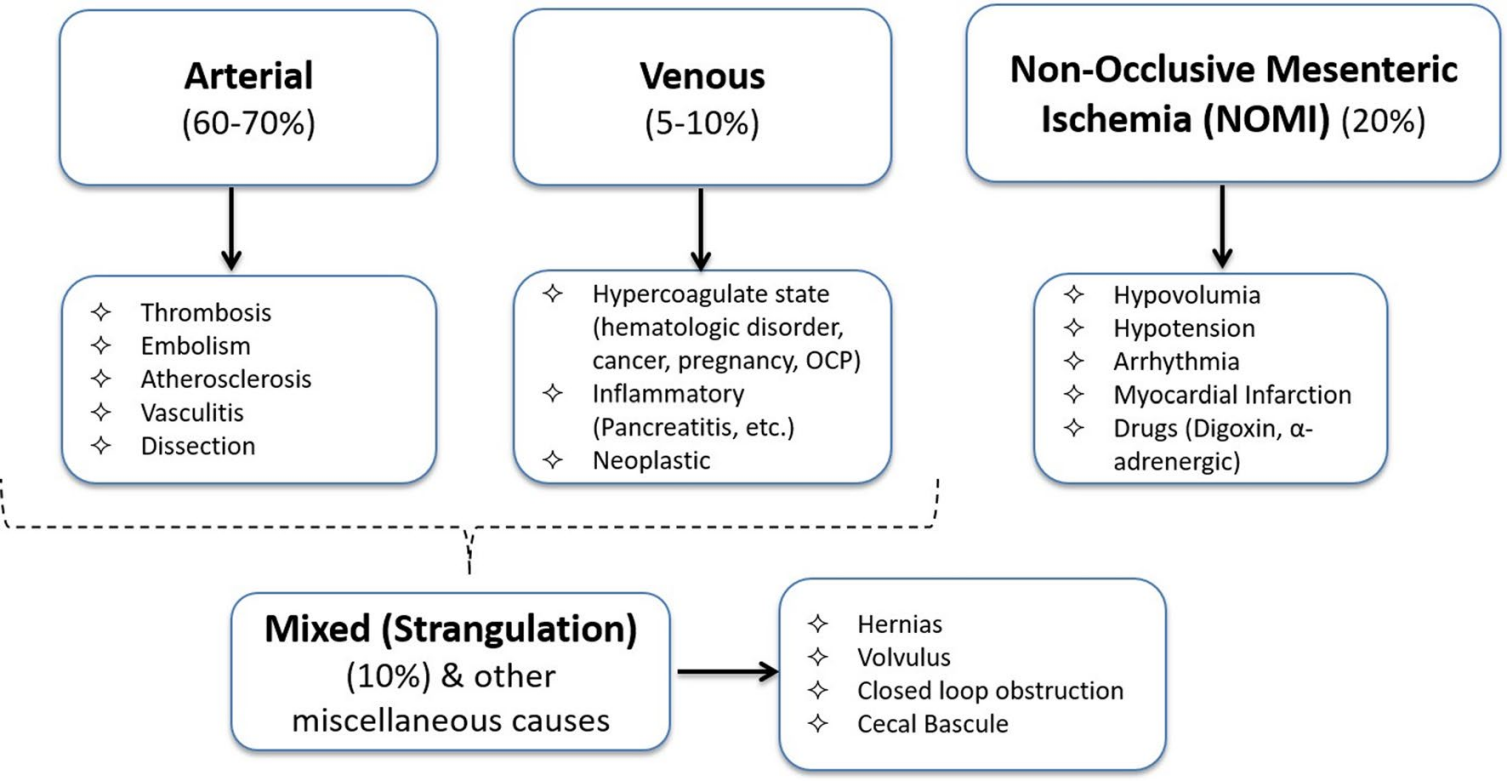
Overview of the causes of acute bowel ischemia

Intestinal ischemia develops when blood perfusion is disproportionate to the metabolic demands of the organ. Collateral circulation can protect the bowel from substantial damage when up to 75% of bowel perfusion is lost for 12 h [14]; however, complete perfusion loss of more than 4–6 h results in significant bowel injury [15]. Therefore, the initial hours are a golden time to achieve a favorable outcome with prompt intervention. ABI has diverse etiologies that could be divided into: arterial, venous, non-occlusive and mixed type [16]. Familiarity with each category will help radiologists take a proactive approach in early diagnosis of these emergent conditions (Fig. 1).

## Mesenteric circulation

**Fig. 2:**
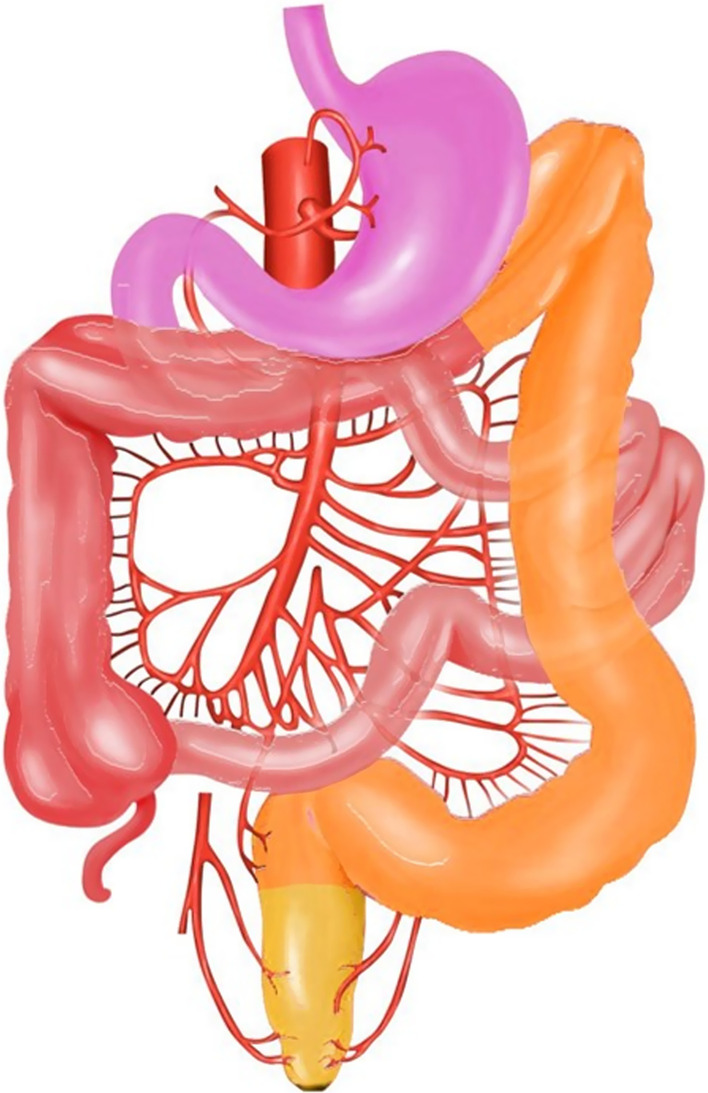
Bowel arterial territories supplied by celiac trunk (pink), SMA (red), IMA (orange) and internal iliac artery (yellow). [Photo was adapted and modified from "Blood supply to the intestines" by Anpol42 (https://bit.ly/3qsQdmY); used under CC BY-SA 4.0]

A solid understanding of vascular anatomy and territories is crucial to identify the areas that are particularly susceptible to infarction from global ischemia. The abdominal aorta gives off three major branches responsible for the arterial blood supply of gastrointestinal tract, namely celiac artery (CA), superior mesenteric artery (SMA) and inferior mesenteric artery (IMA), which perfuse foregut (from the oral cavity to the proximal part of the duodenum, D_1_), midgut (from the mid-duodenum, D_2_, to the proximal two-thirds of the transverse colon) and hindgut (from the distal one-third of the transverse colon to the upper portion of the anus), respectively [17] (Fig. 2). This accounts for the conventional anatomic pattern with other anatomical variations exist [18].

The mesenteric venous drainage includes superior mesenteric vein (SMV) and inferior mesenteric vein (IMV), grossly mirroring the arterial system and running alongside the corresponding arteries. IMV drains blood from rectosigmoid and left colon; meanwhile, SMV drains right and transverse colon, ileum and jejunum. Subsequently, SMV and splenic vein (SV) join together to form the portal vein. IMV can drain into splenic vein (SV), SMV or their confluence [19]. In case of any arterial or venous occlusion, plenty of venous collateral pathways exist.

Absence or underdevelopment of vascular connections between two neighboring colonic vascular territories is regarded as “Watershed” areas. Junction of SMA and IMA at the colon’s splenic flexure is known as “Griffith’s point” [20]. There is an additional watershed area in the rectosigmoid junction between IMA branches and hypogastric arteries known as “point of Sudeck” [21]. These watershed areas are typically affected the most in hypoperfusion state [22].

## Pathologic staging

Understanding the pathologic basis of ABI can help to estimate the probability of reversibility and choose the optimal treatment option. The gastrointestinal tract is made up of four layers, which include mucosa, submucosa, muscularis and serosa, from inner to outermost layer. ABI is categorized into 3 stages based on the involvement of each layer.

In stage I or the early stage, the damage is only confined to the mucosal layer [23]. The early stage of ischemia is typically followed by the release of certain mediators (cytokines, platelet-activating factor and tumor necrosis factor), which leads to an inflammatory response, resulting in further damage of the intestinal wall [24]. These changes are reversible and are not extended to the outer layers. This phase has the best prognosis and can heal completely with timely intervention.

**Fig. 3:**
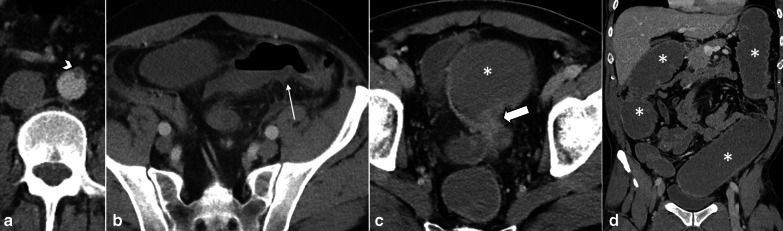
A 48 y/o male presented abdominal distention and diffuse pain. CT images demonstrate complete thrombotic occlusion of IMA (arrowhead) at its origin (**a**). Segmental thickening of sigmoid colon is noted in **b** with hypo enhancement, compatible with watershed ischemia (thin arrow). **c** 3 months later, patient returns with fibrotic stricture (thick arrow) of the distal sigmoid colon with upstream colonic dilatation and obstruction in **d** (asterisk)

**Fig. 4:**
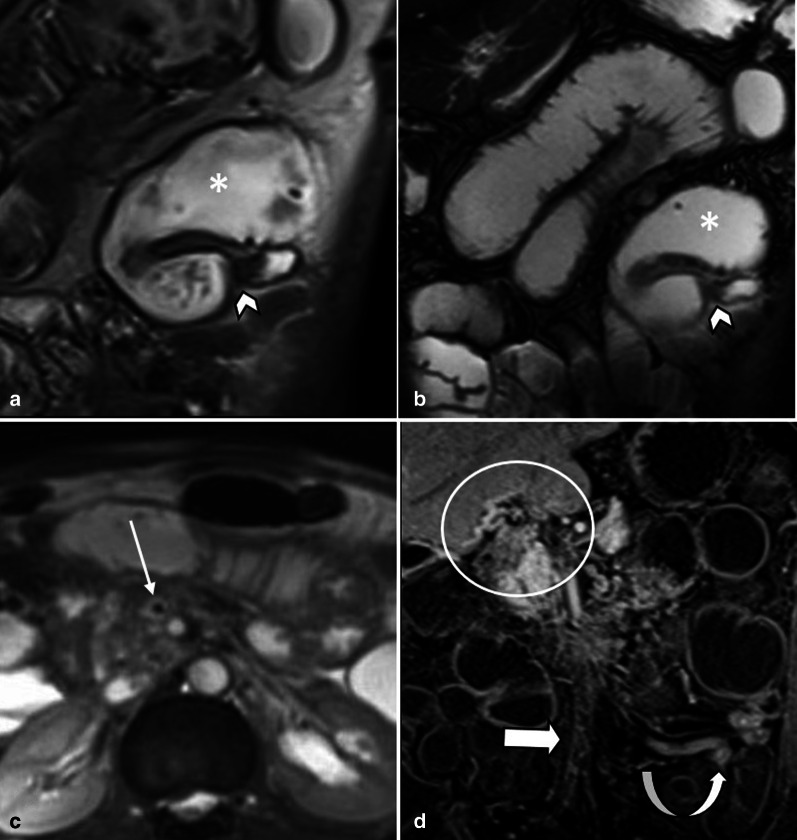
A 44 y/o male with chronic abdominal pain, recurrent episodes of partial small bowel obstruction and long history of portal vein thrombosis. MR enterography images show a fixed stricture (arrowheads) in jejunum on coronal T2-W FSE (**a**) and SSFP (**b**) sequences leading to upstream dilatation (asterisk). A partial filling defect (thin arrow) is also seen in SMV in axial T2-W SSFP sequence (**c**). On the post contrast coronal T1-W sequence (**d**), there are prominent mesenteric collateral structures (curved arrow) in the left hemi-abdomen adjacent to the stenotic jejunal segment. Chronic thrombosis of SMV is seen (thick arrow), with collateral formation (circle) in the porta hepatis due to chronic portal vein thrombosis

Stage II or the intermediate stage is described as presence of specific pathologic changes (edema, erosion, hemorrhage and necrosis) in submucosa and muscularis propria [14]. There are two enteric nervous plexuses in these layers. Meissner’s plexus lies in submucosa and mainly controls secretion and local blood supply, whereas Myenteric or Auerbach’s plexus is located between the longitudinal and circular layers of the muscularis propria and is responsible for the peristaltic movement of the bowel. Injury to these neural plexuses leads to a reduced blood flow, impaired motor function and results in loss of peristalsis and muscles tone [25]. Fibrotic stricture of bowel eventually forms as the late outcome if perfusion restores (Figs. 3, 4).

**Fig. 5:**
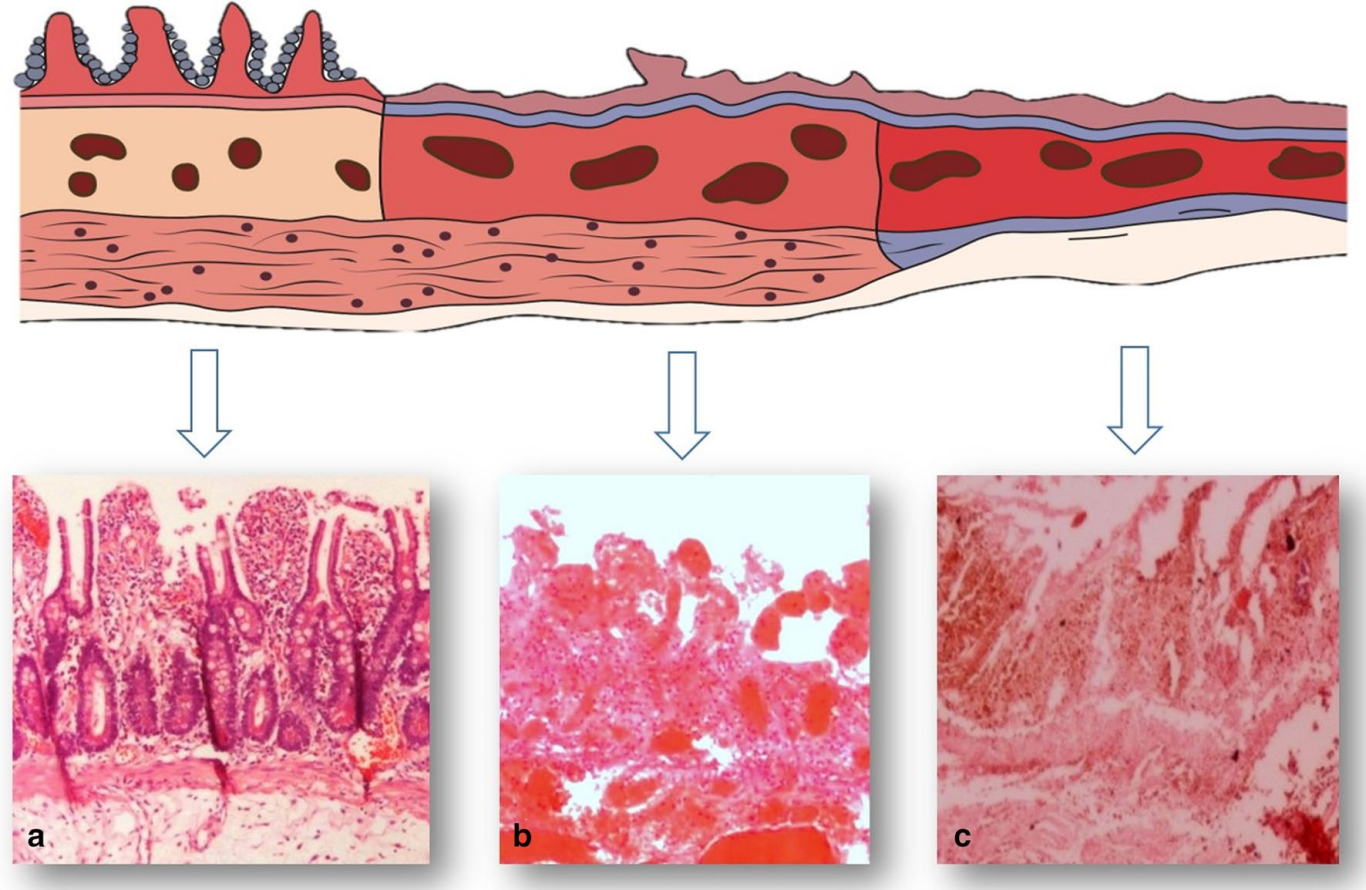
Three pathologic stages of bowel ischemia with corresponding histopathologic samples as early (**a**), intermediate (**b**) and late (**c**). [Top photo reproduced, with permission, from Kumar V, Abbas AK, Fausto N, Mitchell R (editors). Robbins basic pathology, 8th ed. Elsevier, 2007]

Stage III, the late stage, is manifested by transmural bowel necrosis and is developed if blood supply is not eventually restored [23]. Epithelial changes and alterations in cellular function in stage III lead to increased capillary permeability and therefore bacterial translocation [26]. Subsequently, efficient hepatic detoxification is impaired and toxins release into the systemic blood circulation. Consequently, progressive multiorgan failure develops [27]. It should be noted that bowel ischemia accompanied by reperfusion injury may lead to the disruption of mucosal barrier followed by bacterial invasion, septic shock and multiorgan failure, which may cause death without transmural necrosis [28]. This phase is clinically presented with fever, bloody diarrhea and shock. The permanent end-stage bowel damage in this stage accounts for the highest mortality rate and unfavorable prognosis. Therefore, surgical intervention is mandatory [29]. Schematic illustration of pathologic stages in ABI is shown in Fig. 5.

## CT scan protocol

CT images are acquired in supine position from diaphragmatic dome to pubic symphysis covering the entire length of gastrointestinal tract. The protocol consists of unenhanced, arterial and portal venous phases for optimal visualization of hemorrhage, splanchnic vessels and bowel wall. Using unenhanced CT alone is inadequate and results in a delayed diagnosis as well as increased mortality [30]. Following the injection of 2 ml/kg bolus of 350 mg iodine/ml contrast material at the speed of 3–5 ml/s, the scans are obtained in arterial (30 s delay) and portal (65 s delay) phases. For multidetector CT scanners, collimation of 0.5–2.5 mm, detector pitch of 0.9–1.5 and slice thickness of 2.5 mm are used [31]. However, construction of thinner contiguous slices is recommended for multiplanar reformation and CT angiography.

**Fig. 6:**
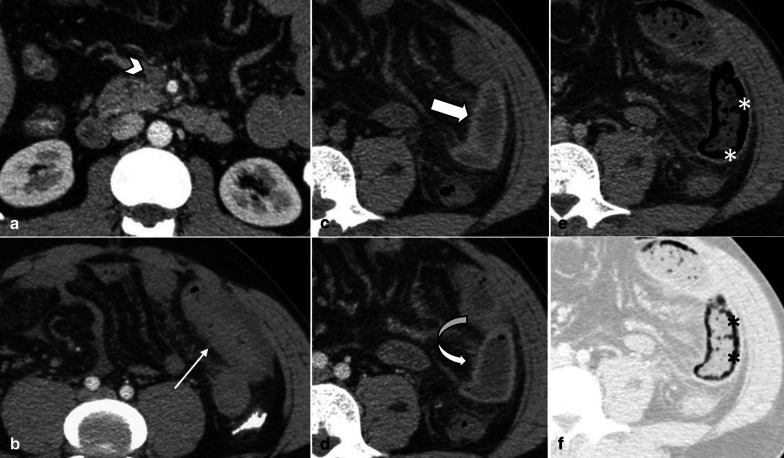
A 32 y/o male presenting with acute SMV thrombosis due to protein C deficiency. CT images on first day of admission (**a**, **b**) display thrombosis (arrowhead) in SMV with segmental circumferential mural thickening in ileum (thin arrow), mesenteric fat stranding and free fluid. On day 3 (**c**, **d**), mural hyperdensity (thick arrow) is noted in non-contrast CT at the same segment without post contrast hyper-enhancement (curved arrow) suggestive of intramural hemorrhage. Mural thickening has relatively decreased. On day 9 (**e**, **f**), mural pneumatosis (asterisks) and thinning (paper thin) are depicted consistent with development of transmural necrosis which was confirmed in laparotomy

Pre-contrast imaging is necessary as some important features of ischemia, like vascular wall calcification, hyperattenuating thrombosis and intramural hemorrhage, might be overlooked in post contrast images (Fig. 6). Oral contrast usage is not routinely recommended for patients suspicious for ABI because of potential delay and obscuration of intraluminal hemorrhage. Furthermore, adynamic ileus secondary to ABI prevents appropriate passage of oral contrast to distal bowel [32]. Biphasic CT scan has also been shown to be adequate for diagnosis of ABI with high sensitivity and specificity [33–38].

## Radiologic staging

Early imaging diagnosis of ABI is often challenging since each individual CT finding is non-specific when observed separately. These include altered bowel wall thickness, attenuation or enhancement along with increased luminal diameter, mesenteric fat engorgement and mesenteric fluid [39]. The late findings suggestive of bowel necrosis include pneumatosis intestinalis, portal venous gas and pneumoperitoneum [40]. CT scan findings have been shown to correlate with pathologic staging and can help to predict the chance of reversibility [28]. Table 1 summarizes radiologic findings of ABI based on etiology and stage.

**Table 1:** Radiologic signs of ABI

General signs	Stage		
	Early	Intermediate	Late
Mucosal hyperenhancement	✓		
Target or Halo appearance	✓		
Hyperattenuating wall (white pattern)	✓	✓	
Mural hypoenhancement (grey pattern)		✓	✓
Thickened bowel wall	✓	✓	
Paper thin wall			✓
Gas pattern			✓
Pneumoperitoneum			✓

Etiology-specific signs	Etiology				
	Emboli	Thrombosis	Venous	NOMI	Strangulation
Calcification of mesenteric arteries		✓			
Completely fluid filled gasless dilated loop			✓		
Ascites	✓	✓	✓✓	✓	✓
Smaller SMA and SMV diameter				✓	
String of sausage sign (in angiography)				✓	
Contrast reflux (in angiography)				✓	
Right colon involvement	✓	✓	✓	✓✓	✓
Beak sign					✓
Fat notch sign					✓

*Early stage:* Bowel wall may be unchanged or thickened at stage I. Edema in submucosa and muscularis propria results in hypoattenuated thickened bowel wall, which is the most common imaging feature with a sensitivity of 38–86% and a specificity of 38–72% [41, 42]. Increased vascular permeability at this stage leads to contrast extravasation and mucosal hyperenhancement, especially in portal venous phase [28]. This mural stratification secondary to the enhancing mucosal and serosal layers with a hypoattenuating layer in between leads to the characteristic “Target” or “Halo” appearance [43]. On the other hand, disruption of arterial blood flow can cause mural hypoenhancement, known as “Pale ischemia,” which has a sensitivity of 62% and a specificity of 96% [39, 40]. Because there is no nerve injury at this stage, bowel lumen maintains normal diameter. Another common appearance is the hyperattenuating wall or “White” pattern. This could be due to intramural hemorrhage at pre-contrast images or increased mural enhancement during reperfusion on post-contrast scans [28]. Reduction of both arterial inflow and venous outflow also results in congestion and prolonged wall enhancement, “shock bowel” [44]. Differentiating a normal enhancing wall from a hyperattenuating wall caused by intramural hemorrhage is impossible without the pre-contrast images. Increased wall thickness, mesenteric fat stranding and ascites are other CT scan findings that are evident in reperfusion injuries, with a high sensitivity but low specificity [41, 45].

*Intermediate stage* Deeper extension of ischemia in stage 2 leads to paralytic ileus and luminal dilatation induced by nervous plexus involvement [46]. This finding has a sensitivity of 39–67% but is relatively non-specific, with a specificity ranging from 29 to 81% [34, 35, 41, 45]. Absence or minimal enhancement in the thickened bowel wall causes the “Grey” pattern at this stage; however, this pattern could also be seen with normal or even thin bowel wall segment [39, 40, 47]. The “Grey pattern” feature has a sensitivity of 40% and a specificity of 88% [41].The late sequel of this stage could be fibrotic stricturing following reparative changes [16].

**Fig. 7:**
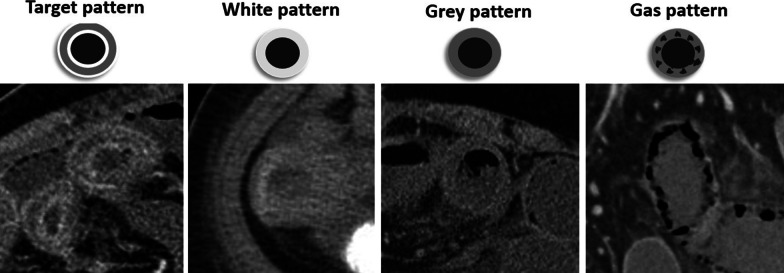
Overview of the enhancement patterns in different stages of bowel ischemia

**Fig. 8:**
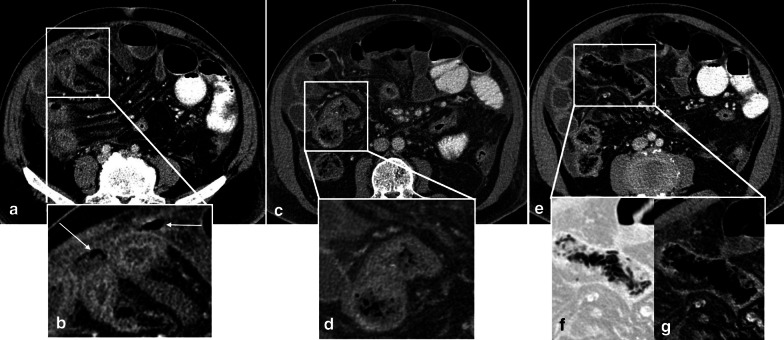
A 68 y/o male with acute SMV thrombosis demonstrates varying degrees of bowel ischemia within the abdomen. **a**, **b** There is increased mucosal enhancement (target pattern) with mural thickening in ileal loops signaling early-stage mucosal ischemia. **c**, **d** Mural thickening and hypo-enhancement are depicted in another ileal segment proximally suggestive of intermediate stage ischemia. **e**–**g** A short ileal segment shows mural thinning, perforation and pneumatosis due to late stage of transmural necrosis associated with pneumoperitoneum (thin arrows in **b**)

*Late stage* In the late stage of arterial occlusion, transmural destruction of nerves and muscular layer leads to dilated, fluid or gas filled loops with extremely thinned wall, creating “Paper thin wall” appearance [48]. Transmural necrosis also causes fluid translocation from intraluminal space to the mesenteric folds, causing mesenteric fat stranding and interloop free fluid as consequences. An additional well-recognized feature for this stage is the “Gas” pattern. This pattern is created as a rim of air bubbles or multiple small separated gas bubbles with circumferential distribution dissect wall layers [49]. Transmural infarct and perforation can lead to translocation of intraluminal gas to the peritoneal cavity (pneumoperitoneum). Overview of enhancement patterns in each stage is shown in Fig. 7. It should be noted that different patterns of enhancement may occur simultaneously in a single patient (Fig. 8).

## Arterial

Arterial etiologies are the most prevalent causes of ABI, accounting for 60–70% cases [50]. Arterial embolus and thrombosis are the most important subtypes.

### Emboli

**Fig. 9:**
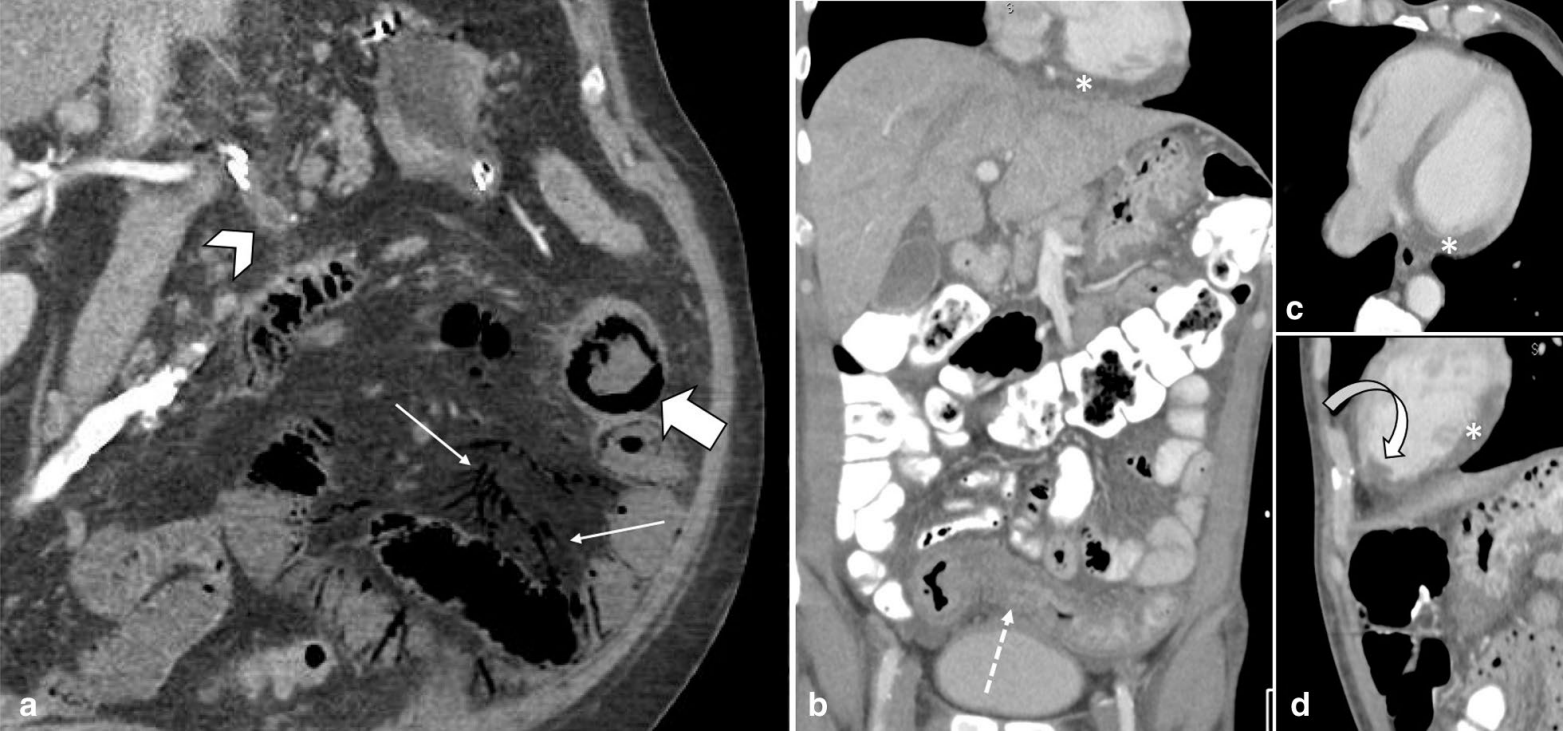
**a** A 52 y/o male with atrial fibrillation presenting with diffuse abdominal pain and guarding. There is a central filling defect (arrowhead) within the SMA approximately 5 cm from the SMA ostium, compatible with acute embolus associated with multifocal pneumatosis (thick arrow) and mesenteric venous gas (thin arrows) consistent with late-stage ischemia. **b**–**d** A 55 y/o male presented with shock and abdominal pain. Incidental transmural left ventricular hypodensity (asterisk) is seen in RCA distribution, compatible with myocardial infarction. Small filling defect is also noted at LV apex, confirmed to represent a thrombus (curved arrow). Bowel wall thickening (dotted arrow) involves watershed region of sigmoid colon, with predominant mural hypoenhancement suggestive of intermediate stage ischemia

Mesenteric arterial emboli (MAE) account for approximately half of all ABI cases [51]. Arterial emboli typically originate from the heart, mainly caused by atrial fibrillation, structural abnormality or ischemic heart disease [52]. SMA is more frequently affected than CA and IMA due to the larger diameter and sharper branching angle (~ 45º) [53]. Considerable narrowing of the SMA immediately after the branching of the middle colic artery creates a vulnerable location for lodging embolus that leads to isolated ischemia of jejunum, ileum and proximal colon with the duodenum and transverse colon usually being spared [54]. The lodgment is also depending on the size of the embolus, with larger emboli tending to occlude proximal mesenteric arteries, resulting in greater extent of ischemia and poorer prognosis [46]. On the contrary, smaller emboli are usually trapped in distal branches causing a limited intestinal involvement with better prognosis [55]. Emboli may also cause end-organ infarctions including lower extremities, spleen, kidneys and brain [43] (Fig. 9).

### Thrombosis

**Fig. 10:**
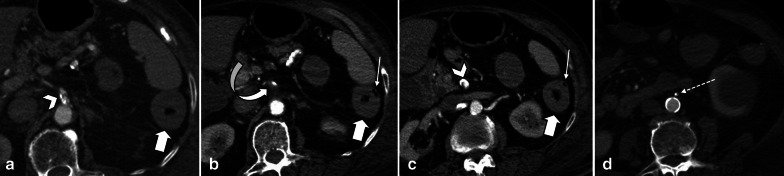
A 78 y/o male presented with severe abdominal pain to ER. Calcified plaques are evident in origin of SMA (arrowhead) and IMA (dotted arrow) leading to notable luminal stenosis associated with superimposed thrombus in SMA. Ischemic changes are depicted in descending colon as circumferential mural thickening and hypo enhancement (thick arrows) along with mural pneumatosis (thin arrows) consistent with late-stage ischemia

Mesenteric arterial thrombosis (MAT) is responsible for 20–35% of ABI cases [51]. Patients generally have long-standing atheroma, or previous stenosis, and have a more indolent course of clinical symptoms [43]. On the unenhanced phase, notable mural calcification of mesenteric arterial branches is usually evident, especially near the ostium and proximal parts of the vessel [56] (Fig. 10). Atherosclerotic changes are typically apparent in other organs including cerebral, renal, coronary and peripheral arteries [57]. The process of ischemia starts when the gradual narrowing reaches a critical level. Then, location and severity of obstruction and development of collaterals determine evolution to bowel necrosis.

CT scan findings for both arterial embolus and thrombosis have great similarities. Low-attenuation filling defect on CT angiography or high attenuation intravascular substance on an unenhanced CT scan is a direct sign for arterial embolus or thrombosis, which can lead to “pale ischemia” and cause “paper-thin” bowel wall [39]. The signs of atherosclerotic changes in the affected artery help differentiate these two entities.

**Fig. 11:**
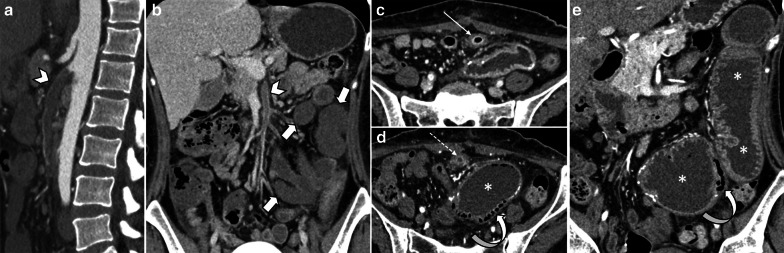
A 33 y/o female with anti-phospholipid antibody syndrome with an extensive SMA thrombosis (arrowheads) and proximal jejunal necrosis (thick arrows) (**a**, **b**). She presented with recurrent abdominal pain 45 days after bowel resection surgery. **c** Marked thickening of small bowel near anastomosis is depicted with mucosal enhancement (thin arrow) suggestive of early ischemic changes. Follow-up CT on day 57 (**d**, **e**) showed dilation of small bowel (asterisk) with pneumatosis (curved arrows) upstream to a late outcome fibrotic stricture (dotted arrow) that was confirmed at surgery

**Fig. 12:**
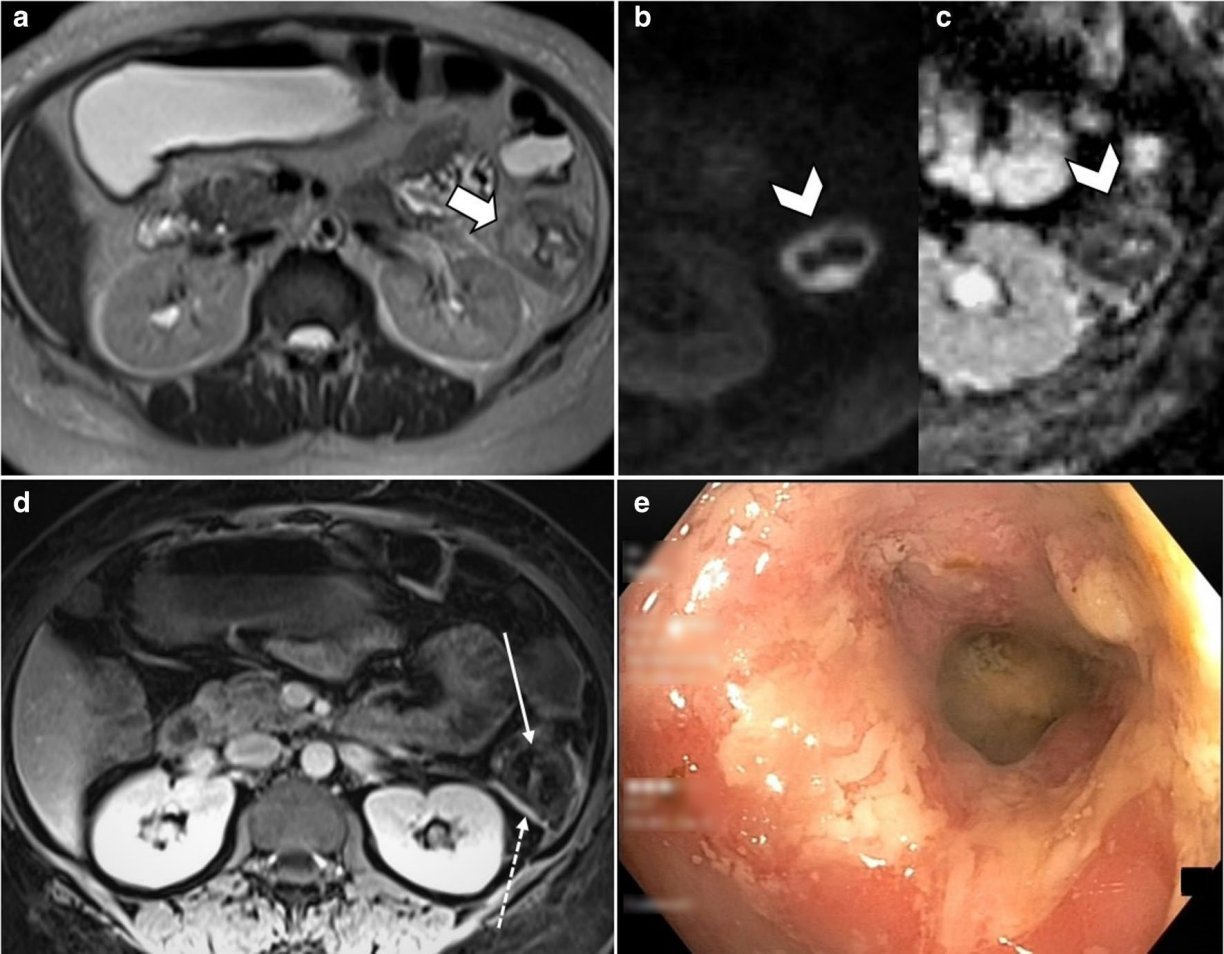
A 37 y/o female with known Systemic Lupus Erythematosus (SLE), presented with abdominal pain and diarrhea. **a** Axial T2-W sequence shows focal circumferential mural thickening (thick arrow) of the descending colon. Intense bowel wall diffusion restriction is noted on DWI (**b**) and ADC (**c**) sequences (arrowheads). On the post contrast axial T1-W image (**d**), there is lack of mural enhancement (thin arrow), favoring Lupus-induced ischemic colitis (intermediate stage). Accompanying mild serosal enhancement (dotted arrow) suggests underlying inflammation secondary to vasculitis. **e** Colonoscopic view of the same segment demonstrated fragile and edematous mucosa with loss of vascularity

Although arterial bowel ischemia has a much higher incidence in the elderly men over 70 years old [58], in younger individuals, thrombotic microangiopathies, antiphospholipid antibody syndrome, dissection and vasculitides may contribute to this type of ABI [16] (Figs. 11, 12). In such cases, unusual sites of the gastrointestinal tract including stomach, duodenum and rectum might be involved.

## Venous

Mesenteric venous thrombosis (MVT) accounts for 5–15% of ABI cases with the youngest peak age of onset and significantly better prognosis when compared to the arterial category (41.7% vs. 73.9% mortality rate) [59]. Although it can be primary on some occasions, the secondary type caused by underlying conditions and hypercoagulability state is much far more common, accounting for 80% of ABI cases [28, 60]. It is typically seen in young females taking hormonal contraceptives [61]. Thrombosis often affects SMV and appears as a filling defect in the portal phase of contrast enhanced CT scan. Sometimes thrombosis could also be depicted as hyper attenuating material in the lumen of mesenteric vein. Combination of vascular findings and bowel wall abnormalities in CT scan resulted in a sensitivity of 96% and a specificity of 90–94% for diagnosis of mesenteric venous ischemia by CT scan [62–64].

**Fig. 13:**
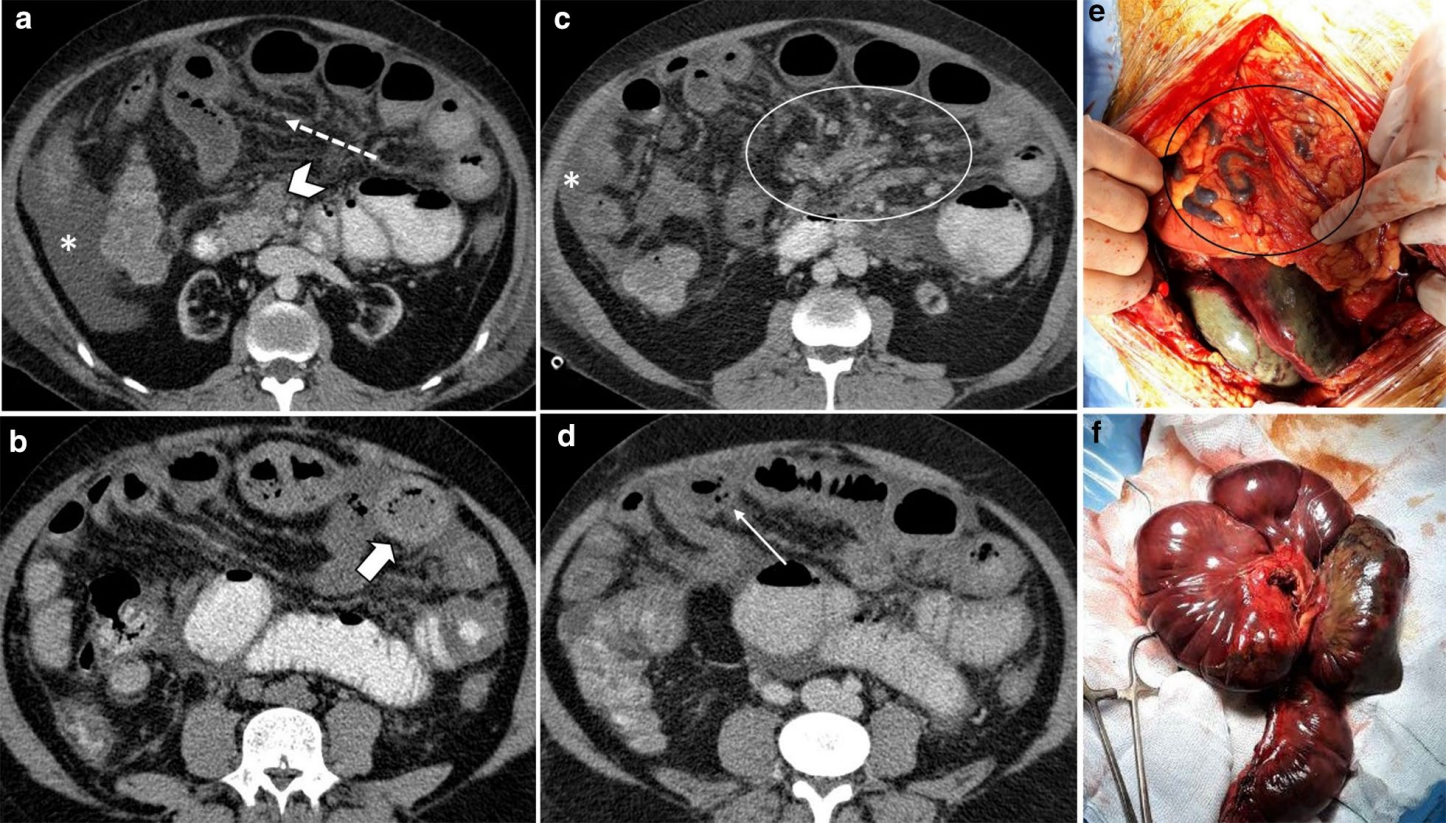
A 54 y/o male with chronic kidney disease undergoing hemodialysis presented with recent abdominal pain and constipation. **a** There is complete SMV thrombosis (arrowhead) leading to extensive ischemic changes in small bowel loops. **b** Hyperdense circumferential mural thickening (thick arrow) secondary to intramural hemorrhage is seen. **c** Mesenteric engorgement is evident (white box). **d** There are also mural pneumatosis, thinning and hypodensity (thin arrow) consistent with late-stage ischemia. Free fluid in abdomen and pelvis (asterisks) and mesenteric edema (dotted arrow) are also depicted. Intra operative images display prominent mesenteric veins (black box) due to engorgement (**e**) associated with small bowel necrosis (**f**)

**Fig. 14:**
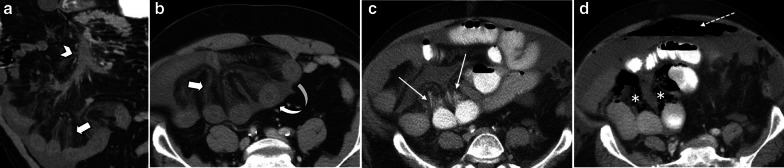
Progression of venous ischemia from early to late stage in a 58 y/o male presenting with abdominal pain. On day 1 (**a**, **b**), baseline Imaging demonstrates extensive SMV thrombosis (arrowhead) with associated small bowel wall (curved arrow) and mesenteric edema (thick arrow), compatible with early stage of ischemia. On day 3 (**c**), follow-up scan demonstrates bowel wall thinning with extraluminal oral contrast (thin arrows) as mesenteric venous contrast intravasation, suggestive of Intermediate stage ischemia. On day 7 (**d**), subsequent CT shows extensive pneumatosis (asterisks) and pneumoperitoneum (dotted arrow), in keeping with late-stage ischemia. Please notice that oral contrast intravasation can precede transmural bowel necrosis

**Fig. 15:**
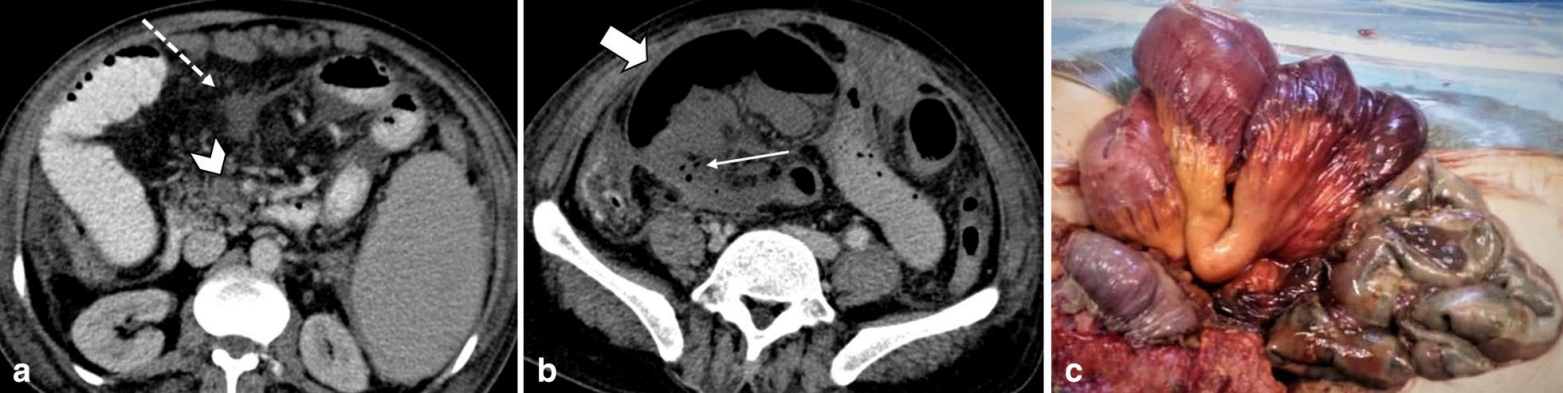
A 66 y/o cirrhotic male patient presenting with acute SMV thrombosis. **a**, **b** CT images reveal complete filling defect in SMV (arrow head) and mesenteric fluid (dotted arrow) associated with mural pneumatosis (thin arrow) and thinning (thick arrow) in a dilated ileal segment. **c** Intra operative image shows necrosis of ileal segments along with some adjacent borderline segments

In the early stage, the bowel wall is frequently thickened [46]. This finding is more prominent than cases of isolated mesenteric arterial occlusion and is the result of reduced venous outflow and maintained high-pressure arterial inflow, which leads to high hydrostatic pressure and intramural edema [40]. “Target” appearance is more common compared to arterial ABI [28, 43]. Wall thickening also can fall out as a result of intramural hemorrhage or superimposed infection may also contribute to the wall thickening in MVT. In veno-occlusive ABI, exudative fluid also accumulates in the intraluminal space of ischemic bowel and results in “completely fluid filled, gasless, dilated loop” [65]. This rarely occurs in arterial form, thus helps in differentiating these entities. Extraluminal seepage of fluid causes mesenteric haziness and ascites [64]. It should be noticed that the degree of wall thickening and ascites is not always proportional to the intensity of bowel wall damage [48] (Fig. 13). Eventually, with persistence of venous outflow occlusion, arterial inflow diminishes leading to decreased or absent wall enhancement showing “Gray” pattern on CT [40]. The findings of the intermediate and late stages are similar to arterial cases (Figs. 14, 15).

Salim et al. demonstrated that small bowel wall edema was the independent risk factor associated with bowel resection in a prospective study on 102 patients with mesenteric venous thrombosis [66]. In a study by Vietti Violi et al. on follow-up of 20 patients with acute mesenteric venous thrombosis, most cases (80%) evolved toward the chronic form with vein stenosis or occlusion and development of collateral veins. It has been reported that patients with short, isolated central mesenteric venous thrombosis in a larger vein might present higher probability of complete radiologic recovery [67]. Higher rate of recanalization in patients with portal or mesenteric vein thrombosis was also observed by Condat et al. compared to patients with more extensive and distal thrombosis [68].

## Non-occlusive

Non-occlusive mesenteric ischemia (NOMI) is responsible for almost 20% of ABI cases [69] and is caused by nonocclusive reduction of arterial blood flow. Hemodynamic instability followed by mesenteric arterial vasoconstriction is the main cause [70], usually seen as a part of systemic hypotensive state associated with impaired sympathetic response in elderly or critically ill patients with concomitant multiorgan damage [71]. CT scan findings have significant overlap with other ABI entities or bowel disorders including infectious and inflammatory diseases of bowel, making the diagnosis even more challenging [72]. The difficulty of early assessment, advanced age, comorbidities and generalized poor physical state are the reasons for the less than 50% survival rate in this subtype [73].

**Fig. 16:**
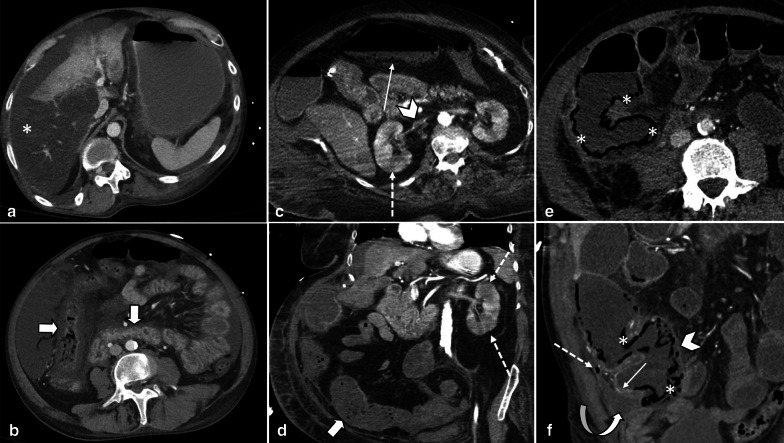
Non-occlusive Mesenteric Ischemia (NOMI) in three different patients. **a**, **b** A 61 y/o male with hypovolemic shock, images show wall thickening, submucosal hypo-enhancement (target wall pattern), and mucosal hyper-enhancement (thick arrows) involving small and large bowel suggesting early-stage bowel ischemia associated with hepatic ischemia as peripheral hepatic hypo-enhancement (asterisk). **c**, **d** A 77 y/o female in shock with bloody diarrhea and renal failure. Colonic distention (thick arrow) with paper-thin wall and subtle pneumatosis (thin arrow). Note collapsed IVC (arrowhead). Renal Ischemia is also seen as heterogeneous delayed nephrogram compatible with acute tubular necrosis (dotted arrows). **e**, **f** A 62 y/o male presented with abdominal distention, pain and sepsis 5 days after CABG. Extensive cecal pneumatosis (asterisks) is noted with hypo-enhancement of medial wall (arrowhead) and hyper-enhancement of lateral wall (thin arrow). There is a rim-enhancing pericecal collection (curved arrow) and extraluminal air (dotted arrow) compatible with perforation and abscess. Patchy areas of necrosis were found at surgery

**Fig. 17:**
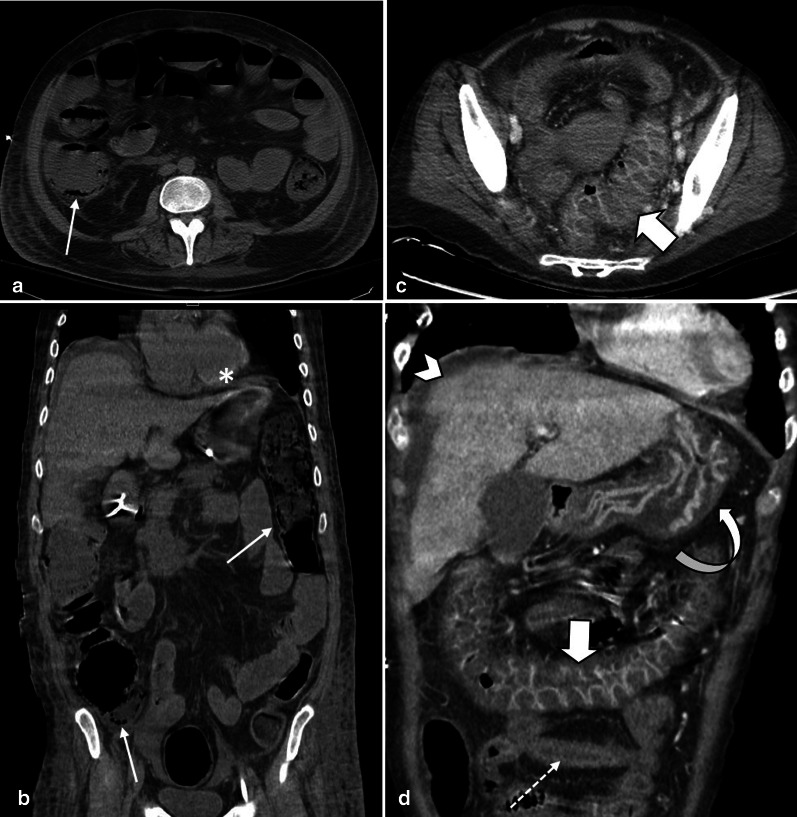
Uncommon manifestations of NOMI in two different patients*.* **a**, **b** A 35 y/o male with history of cardiac transplantation and multiorgan failure. Diffuse bowel distention is seen, with pneumatosis of the colon (thin arrows) and paper-thin colonic wall, suggesting advanced stage of bowel necrosis. Coronal CT image demonstrates dystrophic subtle myocardial calcification in the transplant heart (asterisk) suggestive of rejection. The patient expired from bowel ischemia as a result of cardiac transplant rejection. **c**, **d** A 51 y/o female with alcoholic cirrhosis found to have high anion gap lactic acidosis. Marked diffuse colonic wall thickening is depicted with mucosal enhancement (thick arrow) and “target” wall pattern suggestive of early ischemic injury. Additional involvement of the small bowel (dotted arrow) and stomach (curved arrow). Cirrhotic liver (arrowhead) is also noted

Generalized vasospasm may be obvious as narrowing of IVC, aorta and mesenteric vessels resulting in decreased enhancement of affected bowel segments and occasionally other organs (liver, spleen and pancreas) [74]. Bowel wall thickness ranges from unchanged in earliest stages to markedly thinned in an advanced stage [40, 74]. Radiologic and pathologic findings indicating ischemic staging are the same as other entities (Figs. 16, 17). Four radiological signs had been suggested by Siegelman et al. in 1974 for description of mesenteric vasospasm based on conventional angiography [75]: (1) narrowing in the origins of multiple branches of the SMA, (2) irregularities in intestinal branches as alternate dilatation and narrowing, “the string of sausages sign” [76], (3) spasm of the mesenteric arcades, (4) impaired filling of intramural vessels. Another suggested sign of NOMI has been contrast reflux into the abdominal aorta at angiography [70]. Nakamura et al. reported that diameters of SMA and SMV have been significantly smaller in 11 NOMI cases than 44 controls on CT scan and SMV diameter was a more significant parameter than SMA diameter. Whether these results could be considered as diagnostic criteria remain to be investigated because the so-called smaller SMV sign has also been reported in other etiologies like acute SMA occlusion [77].

During the hypoperfusion state, colon is more vulnerable to ischemia than the small intestine [78]. The anatomic and physiologic characteristics of the mesenteric circulation contribute to particular vulnerability of right colon in classical NOMI. Compared to the left colon, the right colon frequently lacks a well-developed and consistent marginal collateral network. The vasa recta of right colon are usually longer and originating further away from the bowel. Dominant involvement of right colon in NOMI could also be explained by the possibility of "mesenteric steal" from more proximal branches of SMA circulation when perfusion is compromised [79, 80]. Progression of damages occurs in almost a similar manner to small bowel ischemia from mucosa to serosa [81].

## Mixed (strangulation) and other miscellaneous causes

**Fig. 18:**
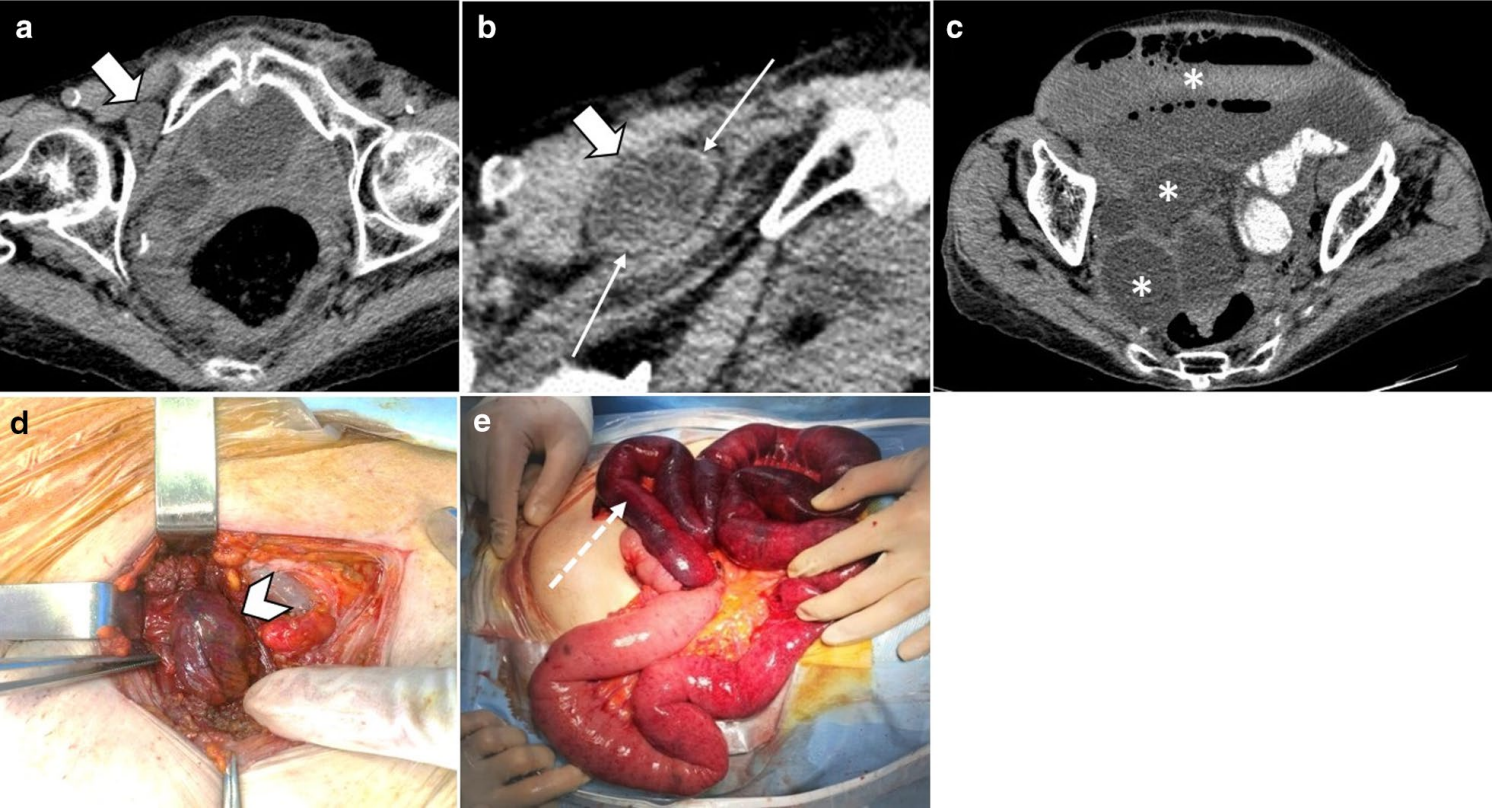
An 84 y/o female with notable abdominal pain and distension for 1 week and obstipation for 2 days. She underwent CT scan with oral and without IV contrast due to elevated serum creatinine level. **a**–**c** Right obturator hernia is seen containing a loop of ileum (thick arrow), causing proximal small bowel obstruction (asterisks). No pneumatosis or pneumoperitoneum was found, so the surgeon decided to make a local inguinal incision to reduce the incarcerated ileal loop; however, this was found to be necrotic (arrow head) (**d**). A subsequent midline incision for laparotomy revealed a long necrotic ileal segment (dotted arrow), that was resected (**e**). Retrospectively, the herniated loop showed high mural density (thick arrow) on non-contrast CT examination suggestive of hemorrhagic infarct

**Fig. 19:**
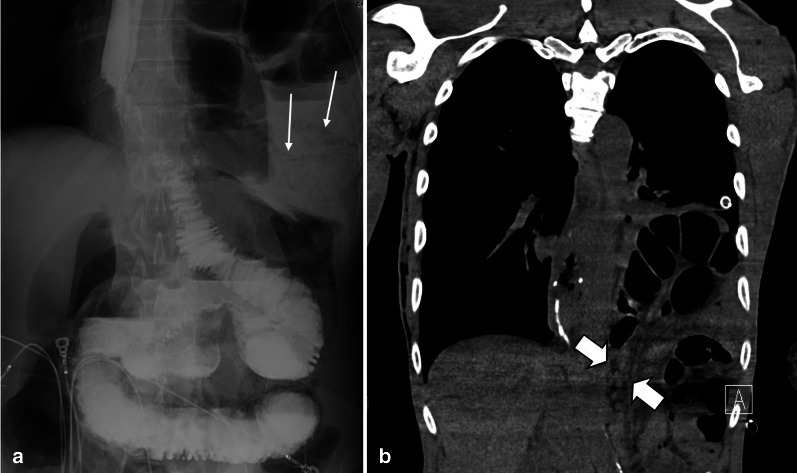
A 59 y/o male status post recent bowel resection for malignancy found to have leukocytosis and abdominal pain. **a** Postoperative left diaphragmatic hernia with pneumatosis (thin arrows) found during postop leak check fluoroscopic exam. **b** Follow-up CT confirmed a diaphragmatic defect (thick arrows) with herniation of bowel loops into the lower chest. Necrotic bowel was found at surgery

**Fig. 20:**
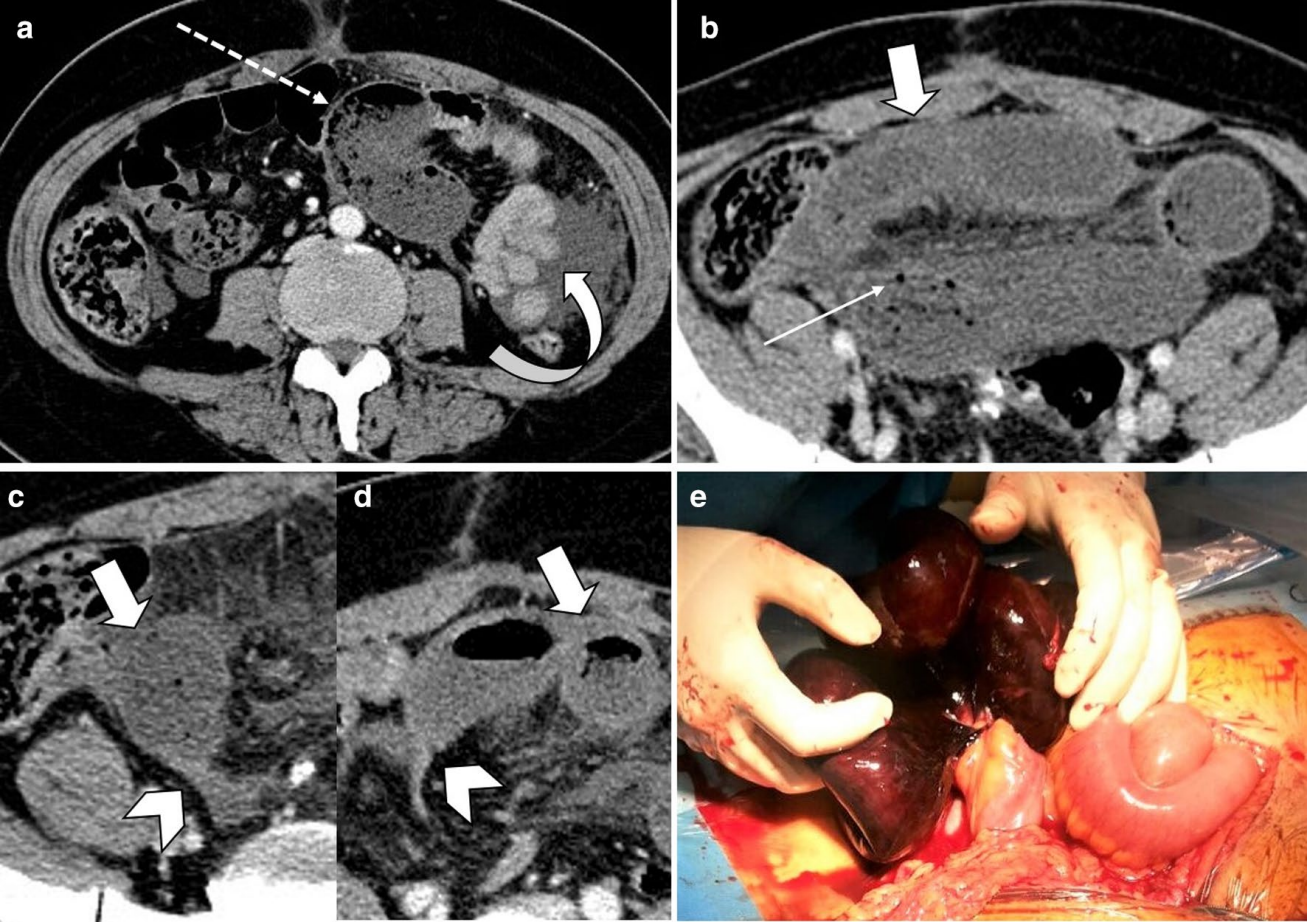
A 45 y/o female with remote prior bowel resection for malignancy presenting with abdominal pain and distension. **a**–**d** Intermediate to late imaging findings of ischemia with dilated small bowel loops with lack of wall enhancement (thick arrows), small bowel-feces sign and minimal pneumatosis (thin arrow), adjacent mesenteric fat stranding and mild interloop fluid. Additionally, the bowel wall is paper-thin (dotted arrow) in the likely necrotic segment with narrowing at two transition points (arrowheads) due to adhesion bands, compatible with closed-loop obstruction. Normal enhancing jejunum (curved arrow) is seen. **e** Corresponding necrotic closed loop of bowel was evident on laparotomy

**Fig. 21:**
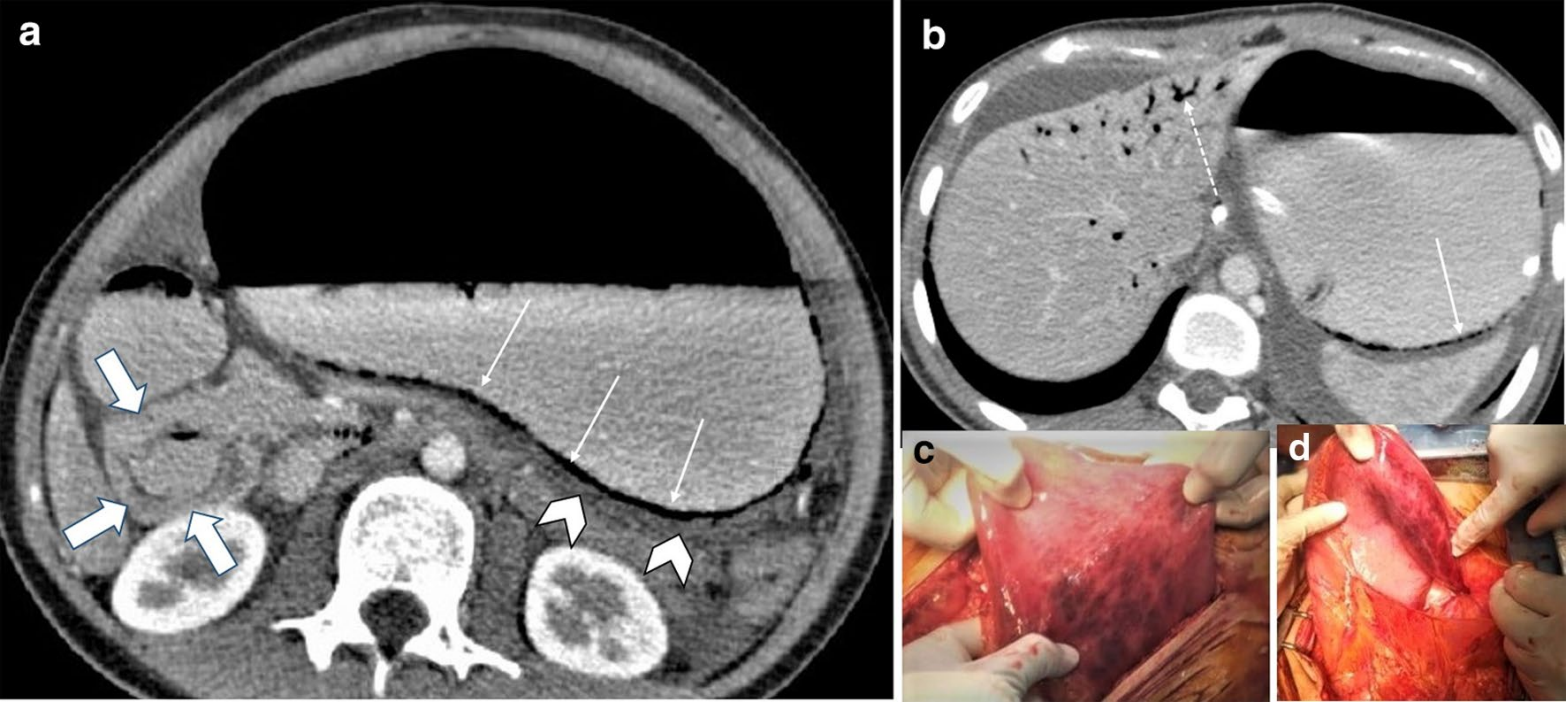
A 17 y/o female with abdominal pain, distension, nausea and vomiting for 3 days. **a**, **b** Marked gastric dilatation is seen with mural pneumatosis (thin arrows) and hypo-enhancement (arrowheads). Portal vein pneumatosis is also depicted (dotted arrow). Patient’s annular pancreas (thick arrows) was causing duodenal luminal narrowing. **c**, **d** Emphysematous gastritis and annular pancreas were confirmed in surgery; total gastrectomy and esophagojejunostomy were performed

Strangulated bowel is usually emanating from closed-loop obstruction and is responsible for 10% of ABI cases [28]. It is described as a twisted C or U-shaped segment of small intestine with both proximal and distal parts obstructed and blood flow compromised, in which the affected segment is dilated, thin walled and fluid filled [82]. Adhesion, hernia and volvulus can also be associated with closed-loop obstruction (Fig. 18, 19) [83]. At earlier stages, arterial input is preserved due to its higher pressure but with taking time both arterial inflow and venous drainage usually get diminished [84]. Signs and staging of ischemia in strangulated bowel are the same as other entities. Some specific features of closed-loop obstruction include radial distribution of intestinal loops and the “beak” sign or triangular loops, caused by fusiform tapering of fluid-filled bowel loops [39, 82]. The closed-loop mechanism in combination with reduced bowel wall enhancement and diffuse mesenteric haziness has been shown to predict strangulation accurately in adhesive small bowel obstruction [85]. Higher risk of ischemia is seen in matted or multiple adhesive bands than single adhesive bands. Zinc et al. have suggested that the beak sign and fat notch sign are associated with the presence of a single adhesive band which can help to personalize patient’s treatment, for example by choosing the laparoscopic approach when non-operative treatment fails [86]. The positive predictive values for a single adhesive band were 92% and 100% for the beak sign and the fat notch sign, respectively [87] (Figs. 20, 21).

**Fig. 22:**
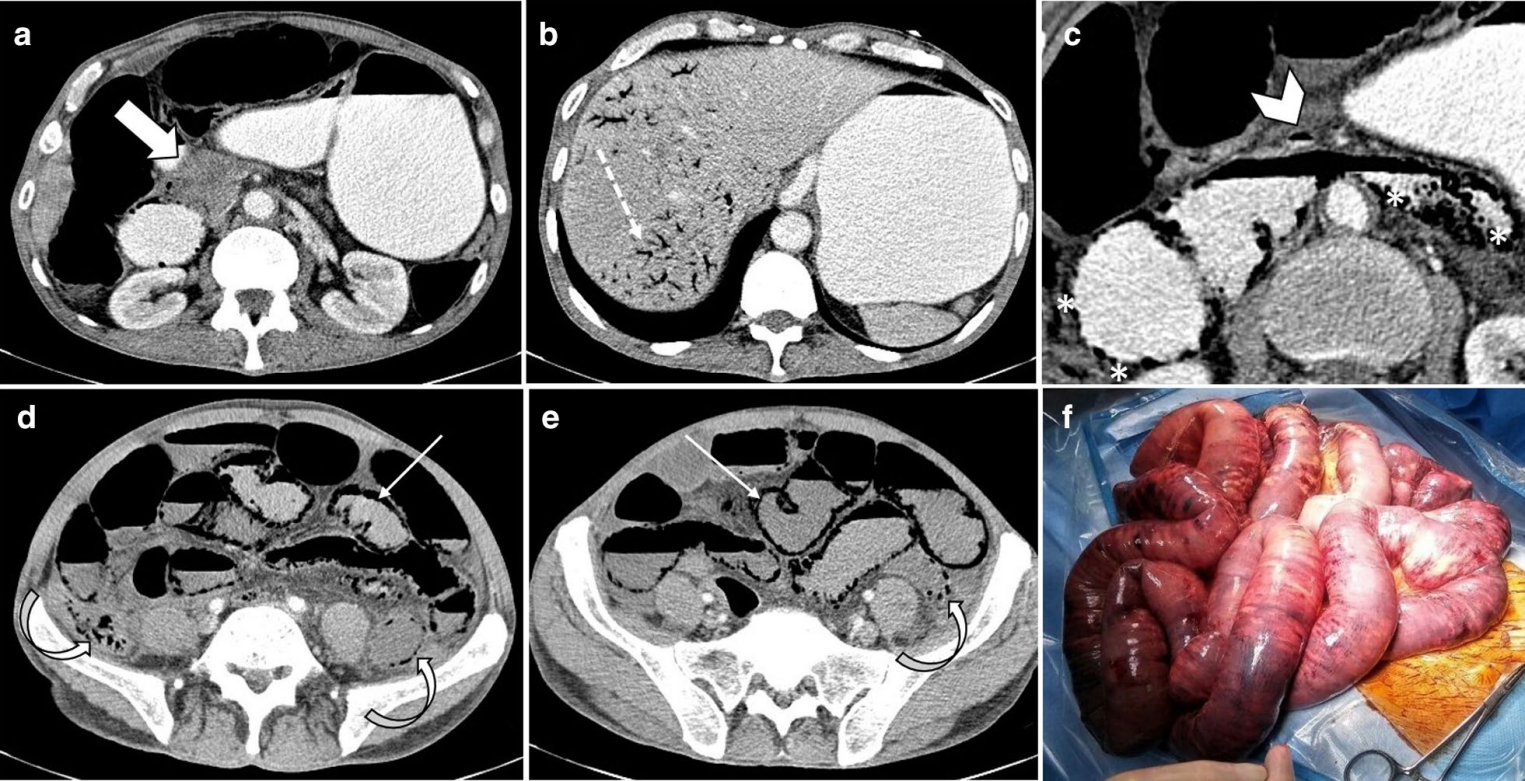
A 68 y/o male with unresectable pancreatic adenocarcinoma with severe abdominal pain and bloody diarrhea 4 days after starting a new chemotherapy regimen with paclitaxel and gemcitabine. **a**–**e** Extensive air is seen in portal vein branches (dotted arrow) and SMV (arrowhead), along with notable intestinal pneumatosis in duodenum (asterisk), small bowel (thin arrows), ascending and sigmoid colon (curved arrow); without a specific mesenteric vascular territorial distribution. Primary tumor is noted in pancreatic head (thick arrow). **f** Bowel necrosis was found in laparotomy from the duodenum to distal colon. No resection was performed due to extensive GI tract involvement, and the patient died

**Fig. 23:**
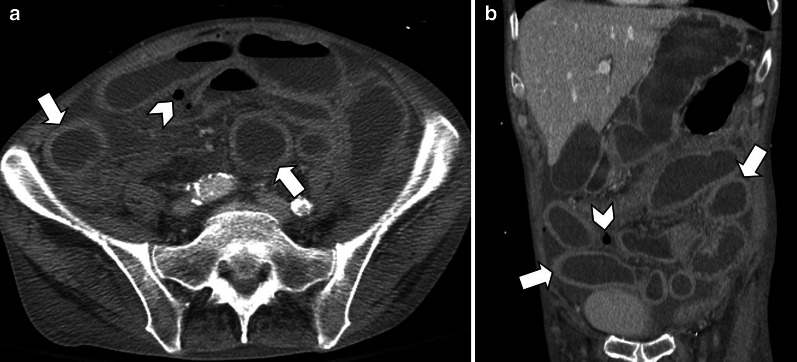
A 64 y/o male with remote history of abdominal liposarcoma, status post whole abdominal external beam radiation therapy. Diffuse marked small bowel wall thickening (thick arrows) is evident involving bowel loops within the radiation field associated with transmural enhancement suggestive of early ischemia. However, there is small volume pneumoperitoneum (arrowheads), due to CT-occult micro-perforation, suggestive of focal bowel necrosis

**Fig. 24:**
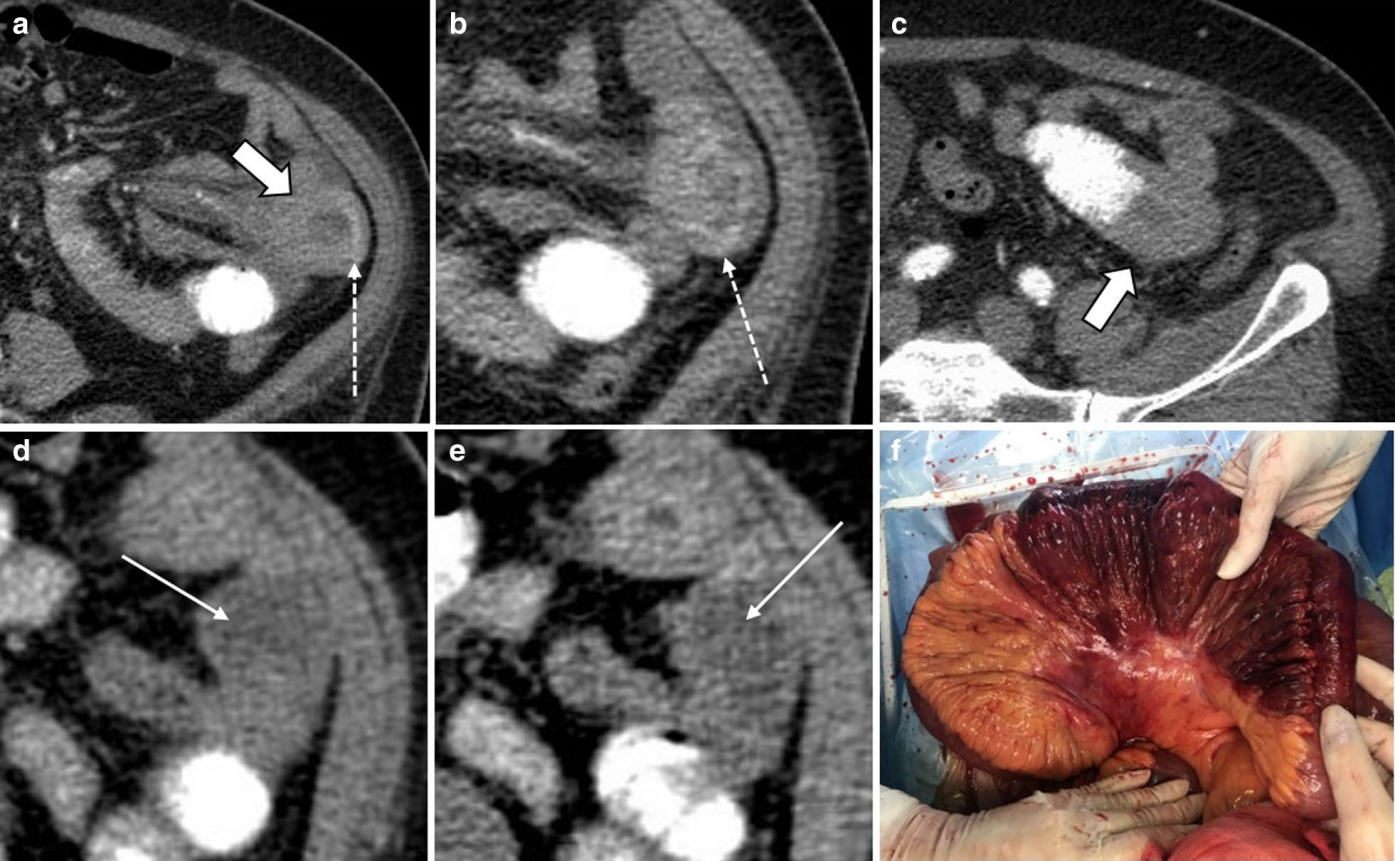
A 68 y/o male with warfarin toxicity was admitted to the ER with left-sided abdominal pain and tenderness for 2 days. **a**–**e** Circumferential mural thickening (thick arrows) with intramural hyperdensity (dotted arrows) is noted in jejunum suggestive of intramural hemorrhage. Additionally, there is an area of hypoenchancing mural thickening (thin arrows) adjacent to the same segment, favoring intermediate to late-stage bowel ischemia. **f** This was confirmed intraoperatively

**Fig. 25:**
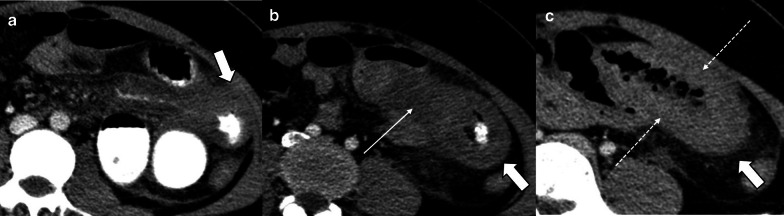
A 27 y/o female ingested ratsbane (rodenticide) in a suicide attempt presenting with abdominal pain and hematochezia. **a**–**c** Segmental circumferential mural thickening (thick arrows) is seen involving the proximal ileum, with adjacent mesenteric fat stranding and mild free fluid. Superimposed focal area of mural hypo-enhancement (thin arrow) (grey pattern) is depicted, suggesting intermediate stage ischemia. There is also transmural mild hyperdensity (dotted arrows) suggesting intramural hemorrhage. Histopathologic results of resected ileal segments revealed mixed transmural necrosis and intramural hemorrhage, presumably due to the warfarin-like effect of ratsbane

**Fig. 26:**
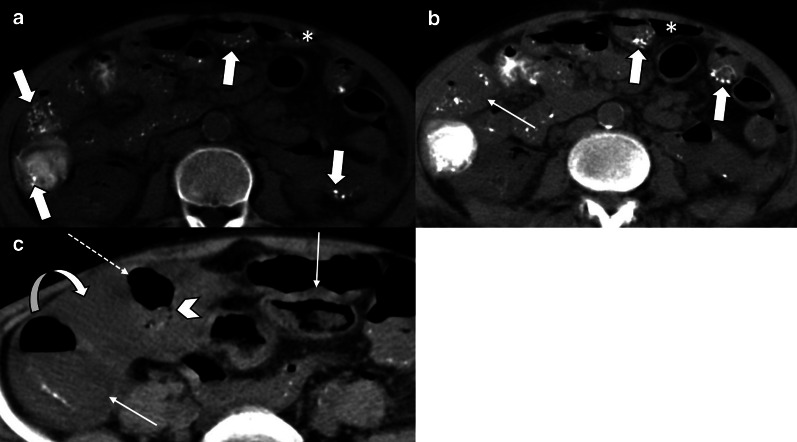
A 56 y/o male with abdominal pain, distension and obstipation for 3 days found to have high serum lead level (> 100 mcg/dl) due to oral intake of lead-contaminated opium. **a**–**c** Non-IV contrast CT revealed intraluminal dense particles (thick arrows) in bowel along with pneumoperitoneum (asterisks), free fluid (curved arrow) and multifocal segmental mural thickening (thin arrows). Additionally, there is a perforation (arrowhead) with adjacent free air, confirmed on laparotomy. Histopathology revealed transmural necrosis in the resected perforated segment

**Fig. 27:**
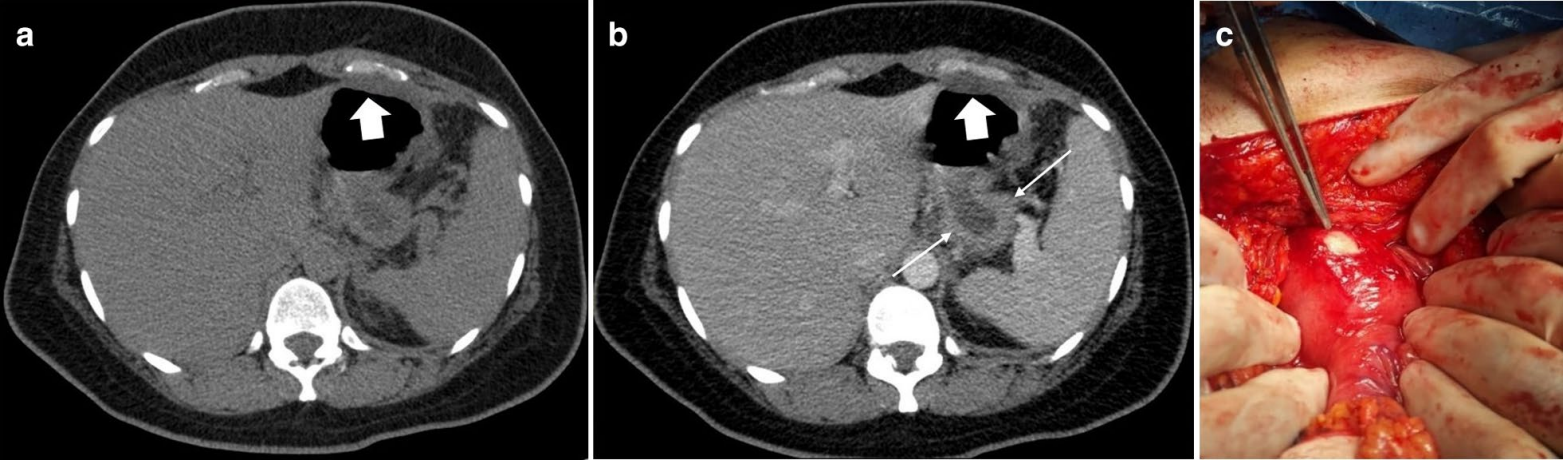
A 42 y/o female with severe epigastric pain 3 days following intragastric balloon insertion for obesity, which was subsequently removed. CT scan was performed 5 days later due to persistent pain and leukocytosis. Pre (**a**) and post (**b**) IV contrast CT scan display focal adhesion of gastric body to anterior abdominal wall with mild mural thickening (thick arrows), demonstrating no enhancement on post contrast images compared to the rest of enhancing gastric wall (thin arrows) (grey pattern). **c** Laparotomy demonstrated anterior gastric wall thinning with pale creamy appearance along with focal perforation due to pressure necrosis from prior balloon placement. The anterior gastric wall was locally resected and repaired

**Fig. 28:**
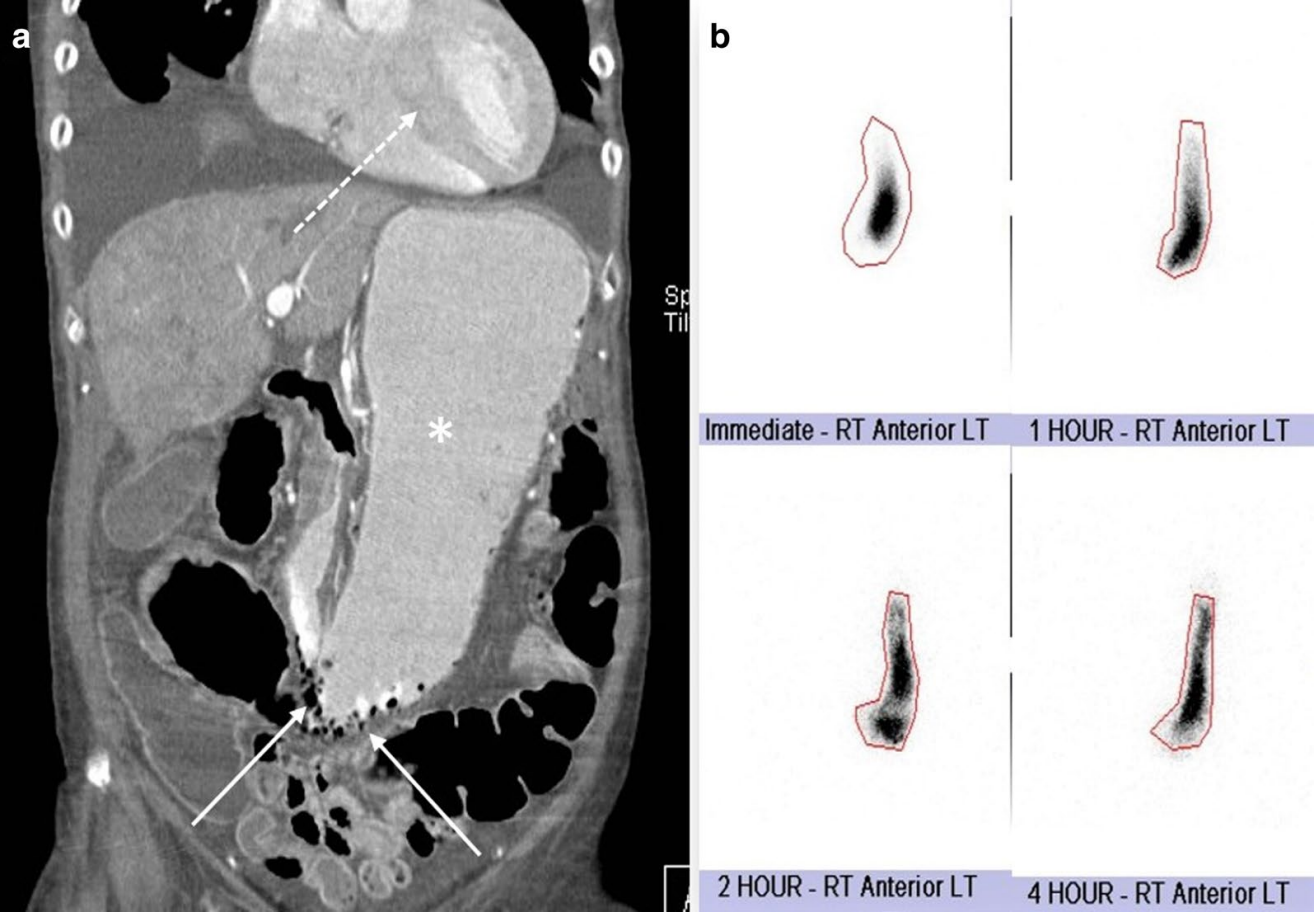
A 56 y/o male with known cardiac amyloidosis, abdominal pain and nausea. **a** CT of abdomen showed thickening of the left ventricle myocardium related to amyloid deposition (dotted arrow). Additionally, there are gastric distension (asterisk) and pneumatosis (thin arrows). Endoscopic biopsy of the bowel wall revealed amyloid deposition as the underlying cause. **b** Prior gastric emptying study showed markedly delayed emptying. Gastric emphysema and ischemia were found at surgery

Mixed arterial and venous ischemia are also infrequently seen in association with other conditions including trauma, chemotherapy, irradiation, drug induced, corrosive injury, lead poisoning, iatrogenic, etc. [74] (Figs. 22, 23, 24, 25, 26, 27, 28).

## Management algorithm

The main goal of management is to achieve a prompt diagnosis, specify the best treatment option and reduce mortality in these patients. ABI is a cardiovascular emergency which entails strict management rules identical to those created for acute myocardial or cerebrovascular ischemia [88]. With immediate management, mortality rate is less than 10%, but this figure increases to 50–60% with moderate delay (6–12 h) and reaches 80–100% with a delay more than 24 h [89]. These results clearly emphasize the close correlation between evolution of necrosis and increasing mortality. Therefore, restoration of the blood flow to the ischemic parts or revascularization is the fundamental principle for surgical and endovascular treatments in all stages. It is worth noting that the morphology of an ischemic bowel can be deceiving and even a severely ischemic bowel can be viable after revascularization [6]. Nevertheless, surgical resection of the necrotic segments is usually inevitable in irreversible stage [90].

The management of ABI is summarized as “3Rs,” Resuscitation, Rapid diagnosis and Revascularization [91]. Invasive hemodynamic monitoring, supportive care, serial assessment of electrolyte level and acid–base status and follow-up imaging are the best approaches in stage I and stage II in which there is no sign of necrosis [3, 6]. Patients should be kept “nil by mouth” which can protect them against ischemic exacerbation and reduce probability of emergency surgery. Systemic anticoagulation therapy with continuous infusion of unfractionated or low molecular weight heparin should be administered as part of early medical therapy especially in cases of veno-occlusive disease, unless there is a contraindication [51, 92]. Broad spectrum antibiotics are also part of initial therapy, especially in critically ill patients [93]. In patients with NOMI, correction of precipitating cause in addition to administration of vasodilators is fundamental [94]. Surgery is established as the best approach in patients with late stage bowel ischemia as revascularization, assessment of organs’ viability and resecting necrotic segments are achievable with surgery [80].

**Fig. 29:**
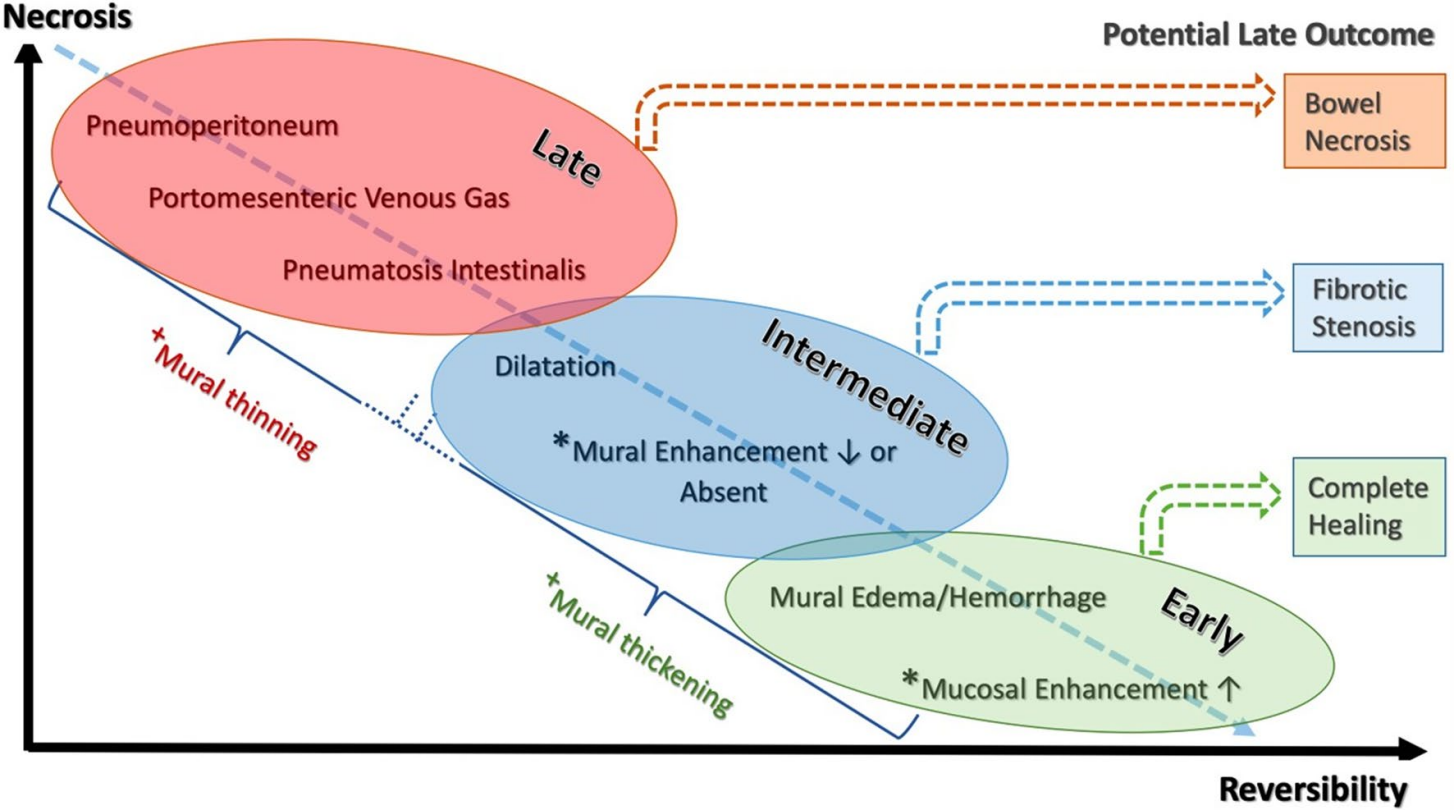
Phases of bowel ischemia from reversible to irreversible with concomitant imaging findings and late outcomes. Note that reversibility–irreversibility spans a temporal continuum with areas of overlap

**Fig. 30:**
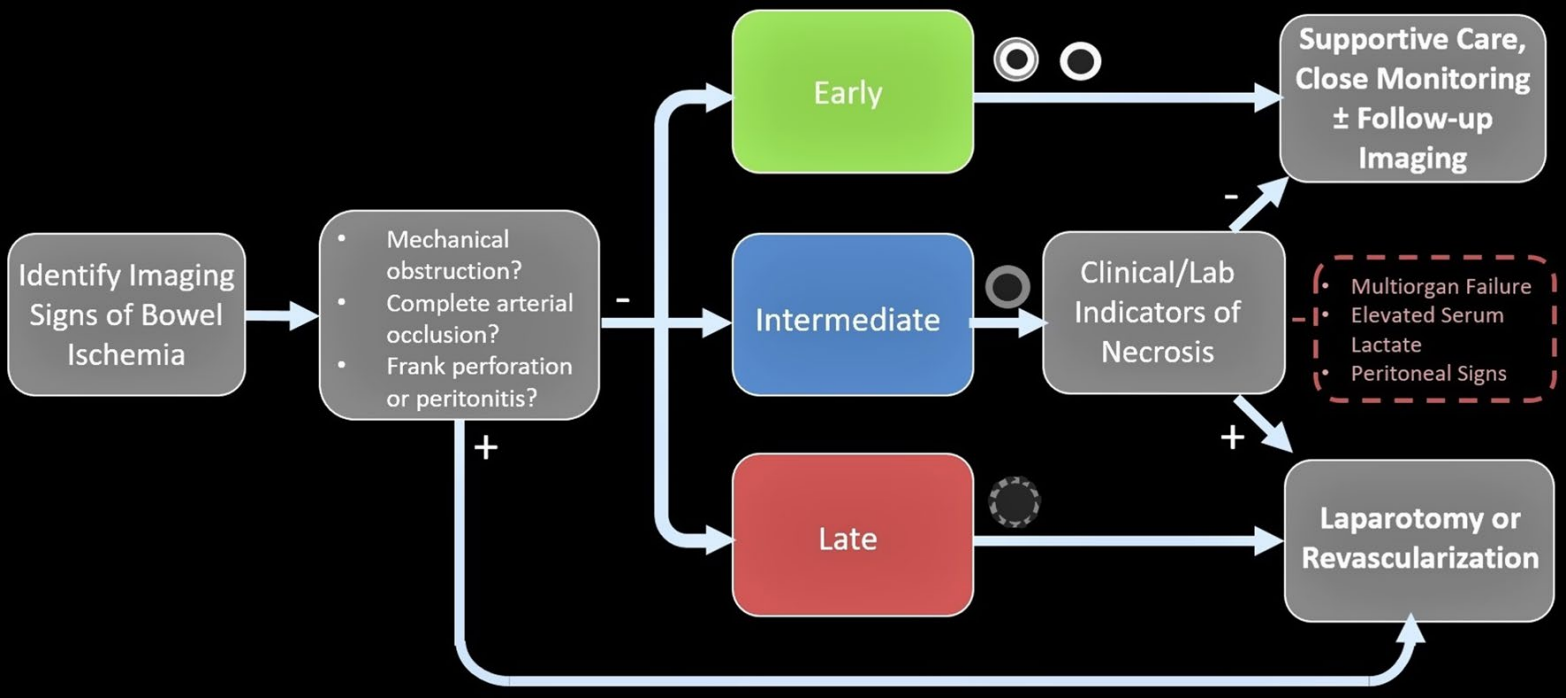
Flowchart depicting management proposal according to radiologic, clinical and laboratory findings

It is noteworthy that reversibility–irreversibility of the bowel spans a temporal continuum with areas of overlap (Fig. 29). In the co-occurrence of stages, management should be based on the latest stage identified. Nuzzo et al. in a prospective cohort of 67 patients with ABI identified three predictive factors of irreversible ischemic injury requiring resection as: (a) organ failure, (b) serum lactate levels > 2 mmol/l and (c) bowel dilation > 2.5 cm at the time of diagnosis. Having 1, 2 and 3 of these factors increased rate of irreversible necrosis to 38, 89 and 100%, respectively. They have suggested that these predictive factors could be useful in emergency setting to decide whether a surgical treatment is mandatory [95]. According to the radiologic, clinical and laboratory findings, a proposed algorithm for management of ABI regardless of etiology is shown in Fig. 30 to determine whether the patient may benefit from surgery. Needless to say, that the goal of surgery is re-establishment of blood supply, resection of non-viable bowel and preservation of viable bowel. According to this algorithm, patients in early stage should be monitored or reimaged, while intermediate stage depends on the clinical and laboratory factors to decide whether the patient needs to be monitored or referred to surgical or vascular intervention. The late stage necessitates rapid laparotomy and/or revascularization.

## Pitfalls

**Fig. 31:**
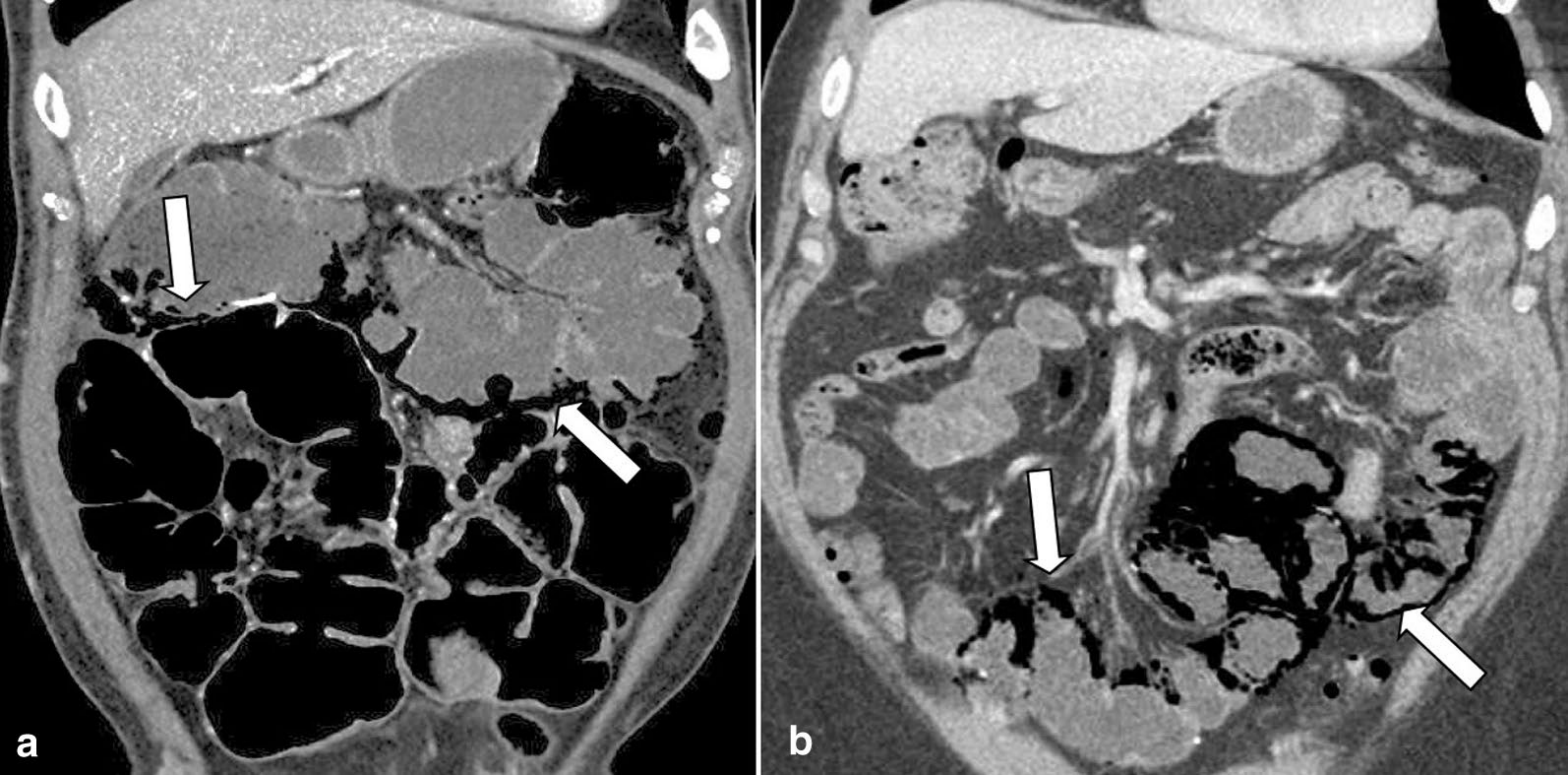
Pneumatosis cystoides intestinalis in two different patients. **a** A 40 y/o male with Crohn’s disease status post ileocecectomy, on corticosteroids. CT imaging demonstrates benign long-segment colonic pneumatosis cystoides (thick arrows) which resolved on 3 months follow-up examination (not shown). **b** A 68 y/o male with abdominal pain. CT shows incidental benign pneumatosis of small bowel (thick arrows), but no other findings suggestive of ischemia. Clinical examinatin was benign, and findings proved to be self-limited on follow-up CT

**Fig. 32:**
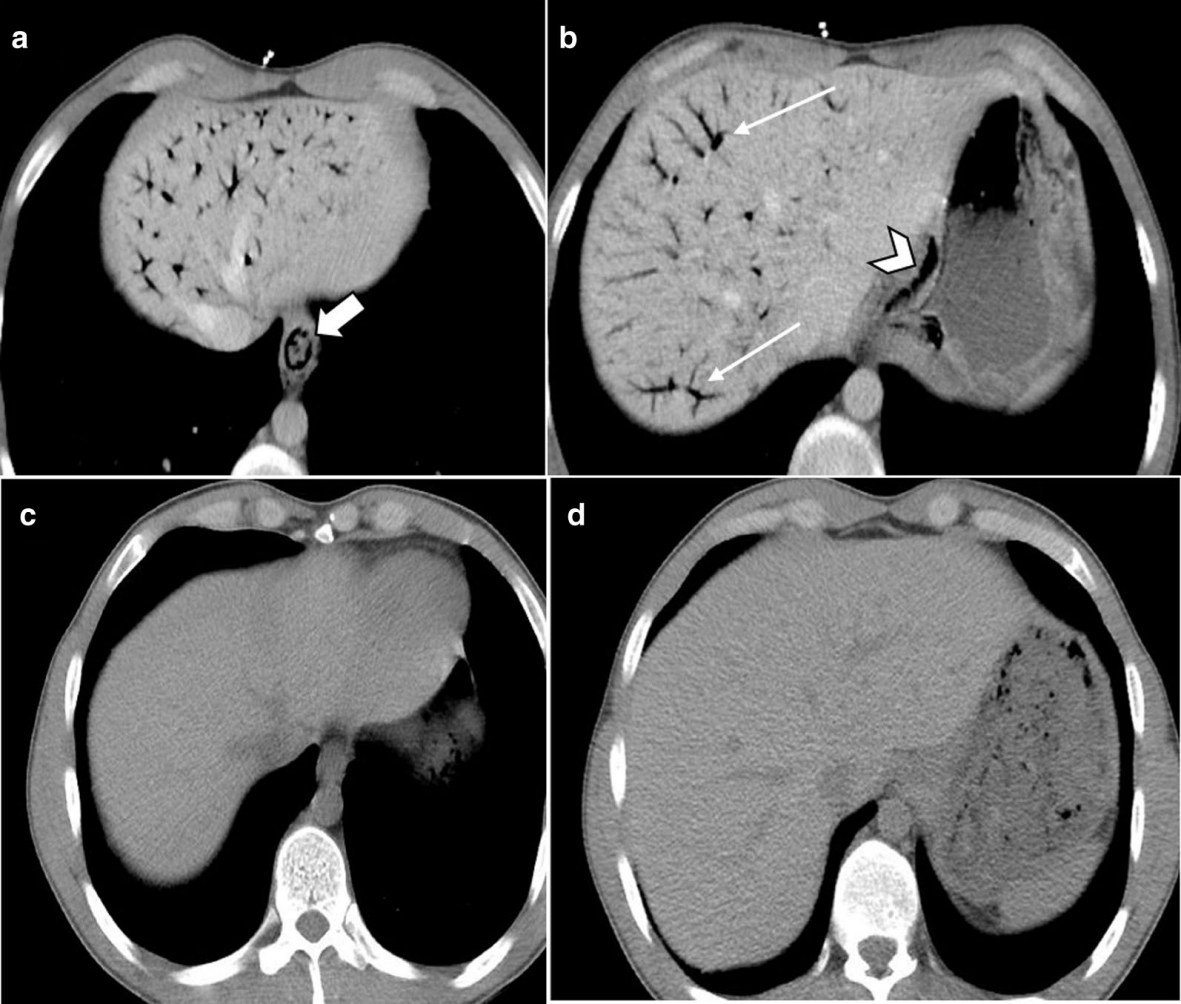
A 23 y/o male presented following hydrogen peroxide ingestion in suicide attempt, found to have transient gastro-esophageal pneumatosis and portal venous gas secondary to esophagogastritis. **a**, **b** Initial contrast-enhanced CT demonstrates intramural air within the distal esophagus (thick arrow) and stomach (arrowhead). There is associated extensive portal venous gas (thin arrows). **c**, **d** Follow-up CT demonstrates interval resolution of portal venous gas, esophageal and gastric pneumatosis. Patient was treated conservatively as there was no evidence of perforation on upper endoscopy

Some non-ischemic conditions may have overlapping imaging features. Most notably, pneumatosis intestinalis may be found in any mechanical mucosal injury, increased mucosal permeability, infections, corticosteroid usage, organ transplantation, inflammation, certain pulmonary conditions with alveolar rupture and pneumatosis cystoides coli [96, 97]. Differentiation of this benign type of pneumatosis from its ominous counterpart as a sign of bowel necrosis might be challenging (Figs. 31, 32).

## Conclusion

Bowel wall ischemia is sub-classified into three pathologic stages depending on the degree of mural involvement (mucosal, submucosal and trans-mural). There are imaging clues that indicate the severity of bowel wall involvement and suggest the pathologic stage. Bowel ischemia may be classified by etiology into the following categories: arterial, venous, non-occlusive mesenteric ischemia (NOMI), mixed arterial/venous and miscellaneous (rare). Imaging provides valuable information regarding the inciting cause. Both the reversibility and imaging stages of ischemia have considerable overlap; however, the management should be based on the latest stage identified. The imaging findings should be interpreted in the context of the clinical picture (serum lactate level, peritoneal signs, multiorgan failure, etc.) and management decisions should ultimately be guided clinically. Decision regarding whether the patient needs surgery or not is the most important question from clinician, which is usually unrelated to the etiology of the ABI since any cause of bowel ischemia may eventually results in a single outcome that is bowel necrosis. Therefore, focusing on the predictive factors of bowel necrosis rather than etiology can better help on the management of ABI cases.

## Data Availability

Data sharing is not applicable to this article as no datasets were generated or analyzed during the current study.

## Abbreviations

ABI: Acute bowel ischemia
CA: Celiac artery
IMA: Inferior mesenteric artery
IMV: Inferior mesenteric vein
MAE: Mesenteric arterial emboli
MAT: Mesenteric arterial thrombosis
MDCT: Multidetector computed tomography
MVT: Mesenteric venous thrombosis
NOMI: Non-occlusive mesenteric ischemia
SMA: Superior mesenteric artery
SMV: Superior mesenteric vein
SV: Splenic vein
